# Isoflavones in Animals: Metabolism and Effects in Livestock and Occurrence in Feed

**DOI:** 10.3390/toxins13120836

**Published:** 2021-11-24

**Authors:** Dino Grgic, Elisabeth Varga, Barbara Novak, Anneliese Müller, Doris Marko

**Affiliations:** 1 Department of Food Chemistry and Toxicology, Faculty of Chemistry, University of Vienna, Währinger Str. 38-40, 1090 Vienna, Austria; dino.grgic@univie.ac.at (D.G.); elisabeth.varga@univie.ac.at (E.V.); 2 BIOMIN Research Center, Technopark 1, 3430 Tulln, Austria; barbara.novak@dsm.com (B.N.); anneliese.mueller@dsm.com (A.M.)

**Keywords:** isoflavones, genistein, daidzein, glycitein, animal feed, pigs, ruminants, poultry, zearalenone, mycoestrogens, phytoestrogens

## Abstract

Soybeans are a common ingredient of animal feed. They contain isoflavones, which are known to act as phytoestrogens in animals. Isoflavones were described to have beneficial effects on farm animals. However, there are also reports of negative outcomes after the consumption of isoflavones. This review summarizes the current knowledge of metabolization of isoflavones (including the influence of the microbiome, phase I and phase II metabolism), as well as the distribution of isoflavones and their metabolites in tissues. Furthermore, published studies on effects of isoflavones in livestock species (pigs, poultry, ruminants, fish) are reviewed. Moreover, published studies on occurrence of isoflavones in feed materials and co-occurrence with zearalenone are presented and are supplemented with our own survey data.

## 1. Introduction

**Figure 1:**
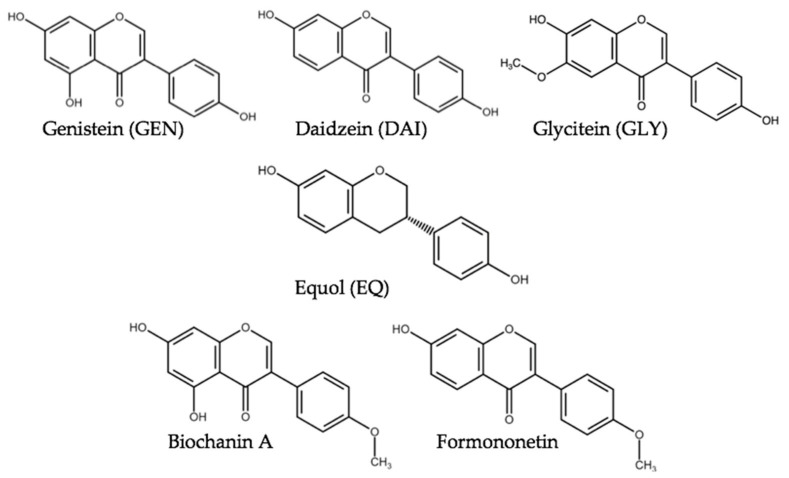
Structure of various isoflavones and equol, a microbial metabolite of daidzein.

Isoflavone(s) (ISF) are secondary plant metabolites belonging to the group of polyphenols [1]. They are characterized by the presence of a benzene ring attached to the third position of the carbon ring [2]. Due to structural and functional similarity with the estrogen-active hormone 17β-estradiol, ISF are classified as phytoestrogens [3]. ISF (Figure 1) are mainly found in plants of the legumin family and thus, high concentrations occur in soybeans (*Glycine max*) and red clover (*Trifolium pratense*). They reside as glycosides with low estrogenic activity compared to their deglycosylated form also referred to as aglycone. Upon ingestion, these compounds are metabolically hydrolyzed by the intestinal microflora to their aglycones thus potentially mediating an estrogenic stimulus [2,4].

Soy products are commonly used as animal feed and due to the increased global production and high protein content of about 38%, soybeans are the most favored protein provider for pig feed [5]. Soybean-based products contain primarily daidzein (DAI), genistein (GEN) as well as their conjugates and in small amounts glycitein (GLY). The overall ISF content of soybean is 1.2–4.2 g/kg dry weight according to older analysis, which agrees with a recent study where the average amount of ISF varies between 0.7 and 5.2 g/kg [6,7]. In the diet of livestock, besides soybeans also red clover is one of the main sources of ISF. The main ISF in red clover are formononetin and biochanin A [1] and ISF occur in concentrations as high as 10–25 g/kg of the dry weight [8] with formononetin accounting for 0.8–11 g/kg of the dry weight [9]. ISF concentrations depend on the plant part, growth stage, cultivar, growing conditions, and preservation method [10,11].

Over the last 50 years, interest in ISF and their effects on animals has increased, especially in the field of agricultural research [12]. Studies have been conducted in various animal species including pigs, cattle, sheep, poultry, and rodents, which found positive as well as negative effects of ISF on the animal health [13,14,15,16,17,18,19,20,21,22,23,24,25]. Positive properties that were attributed to ISF are its growth-promoting, antioxidant and antimicrobial effects [26]. However, negative effects of ISF on the reproductive tract were observed in animals, including symptoms such as an enhanced rate of endometriosis, inability to become pregnant and abortions [27]. The first reports of negative effects of ISF on reproductive health date back to the 1940s, when reproductive problems were observed in sheep after excessive consumption of ISF-containing *Trifolium subterraneum* (subterranean clover), a condition that became known as “clover disease” [28].

**Figure 2:**
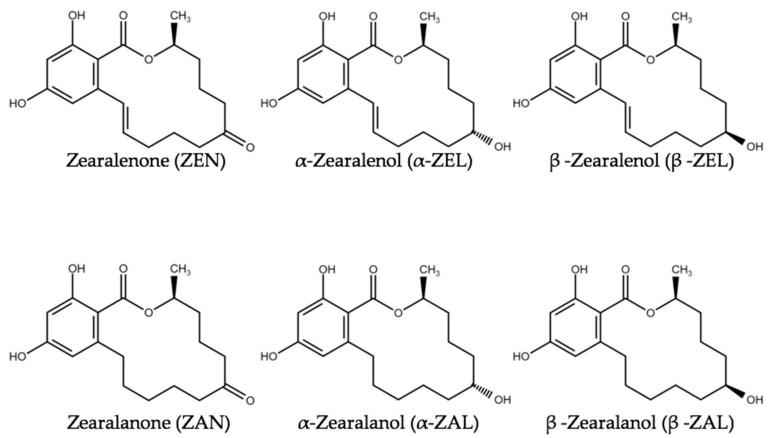
Structure of the mycotoxin zearalenone and its phase I metabolites.

In addition to phytoestrogens, other feed contaminants such as mycotoxins possess estrogenic potential. Estrogenic mycotoxins are termed “mycoestrogens”. The most prominent examples of mycoestrogens are zearalenone (ZEN) and its derivatives (e.g., α-zearalenol, β-zearalenol, zearalanone, α-zearalanol, and β-zearalanol) (Figure 2). ZEN and its derivatives belong to the group of macrocyclic resorcylic acid lactones and are typically formed by molds of the genus *Fusarium* (e.g., the species *F. graminearum*, *F. monoliforme* and *F. culmorum*) [29,30]. Adverse effects of ZEN in farm animals were extensively described [31,32,33]. The most frequently described property of ZEN is its estrogenicity. Pigs are most susceptible to those effects [30,34]. In addition, genotoxic, carcinogenic and immunotoxic effects of ZEN were reported [30] Therefore, the European Commission published guidance values for ZEN in feed that amount to 100, 250 and 500 µg/kg for piglets, sows and calves/dairy cattle, respectively [35,36]. These values were exceeded in some samples of maize destined to be used as animal feed [37,38,39].

Since both mycoestrogens and phytoestrogens can occur simultaneously in feed [40], combinatory estrogenic effects might be possible. Vejdovszky et al. showed estrogenic effects of ZEN and GEN depending on the used ratios and concentrations in vitro [41]. However, further data on the combinatory effects of phyto- and mycoestrogens are lacking.

This current review summarizes the metabolization of ISF including the influence of the microbiome, phase I and phase II metabolites as well as the distribution and inter-individual variability. Furthermore, in vivo studies on effects of ISF in farm animals are reviewed. The focus is on pigs, poultry, and ruminants. In addition, effects in fish are summarized briefly. Furthermore, published data on the occurrence of ISF in feed and co-occurrence with mycoestrogens are presented and supplemented by occurrence data obtained during our own studies.

## 2. Metabolism of ISF

### 2.1. Influence of the Microbiota

The intestinal microbiome plays an important role in the digestion of various food components. Bacteria that colonize the digestive tract are known to modify ISF, which are represented in plants as both glycosides and aglycones. Before ISF can be absorbed from the gut, the sugars of the glycosides must be deconjugated by β-glucosidases expressed by intestinal bacteria and subsequently, ISF enter the bloodstream via passive absorption [42,43,44]. Mammalian β-glucosidase activity does not appear to substantially contribute to deconjugation of ISF glycosides in monogastric animals due to its lower expression level [45].

Apart from the deglycosylation of ISF, further metabolic conversions of ISF are catalyzed by the gut microbiome. In vitro, metabolism of GLY by monogastric fecal flora starts with demethylation to 6-hydroxy-DAI, followed by reduction to 6-hydroxy-dihydro-DAI and subsequent metabolization either by cleavage of the C-ring to 5′-hydroxy-*O*-demethyl-angolensin or by maintenance of the C-ring to 6-hydroxy-equol [46].

Biochanin A can be demethylated to GEN by the gut microbiome, both in ruminants and in monogastric animals. GEN is then further processed by the intestinal microflora in different ways depending on the animal species [47,48]. In ruminants, GEN is degraded to *p*-ethyl phenol and organic acids by ring opening [48]. In monogastric animals, GEN is reduced to dihydro-GEN by the intestinal microflora and further metabolized to 6-hydroxyif-*O*-demethyl-angolensin (6-OH-ODMA) [47].

Analogous to biochanin A, formononetin is demethylated to DAI and subsequently reduced to dihydro-DAI (DHD), which is then either degraded by cleavage of the C-ring to *O*-demethyl-angolensin (ODMA) or the formation of equol (EQ) is initiated while retaining the C-ring. This metabolic degradation of DAI is observed in all livestock species [48,49,50]. However, the capacity of EQ formation varies between the different species. Ruminants possess gut microbiota that favor the biosynthesis of EQ and therefore all ruminants are considered EQ producers [51,52], whereas in pigs the capacity to form EQ is limited and only pigs with certain bacterial strains are classified as EQ producers [53].

The different ability to produce EQ between species is due to the dissimilar composition of the gut microbiota and can also vary with age. The metabolic conversion of DAI to DHD and further to EQ was observed by bacterial strains SNU NiuO16 and SNU Julong732 isolated from bovine rumen [54]. In addition, bacterial strain *Slackia* sp. D-G6 isolated from chicken intestines was described to produce EQ [55]. Similarly, *Eubacterium* strains D1 and D2 isolated from pig feces also possess the ability of this metabolic conversion of DAI. However, these isolated strains showed a lower metabolic conversion to EQ than complex mixtures of different fecal bacteria, indicating that other bacterial species are also involved in the formation of EQ [56].

### 2.2. Phase I Metabolism

After absorption, ISF undergo further metabolic processes in the intestine and liver. During phase I metabolism, oxidative modifications by cytochrome P450-dependent monooxygenases (CYP) occur. In vitro, GEN and DAI are converted by rat liver microsomes to different mono-, di-, and trihydroxylated compounds [57]. Biliary and intestinal CYP isozymes (e.g., 1A1, 1A2, 1B1, 2E1 and 3A4) involved in hydroxylation of GEN were already identified in the late 1990s [58]. A similar metabolic pattern was shown for EQ in microsomes of the same species. EQ was metabolized to mono- and dihydroxylated compounds, with 3’- and 8-hydroxyequol being the major products [59]. In a similar experiment, GLY was mainly transformed in rat liver microsomes to two monohydroxylated GLY derivatives and the demethylation product 6-OH-DAI. These results are supported by in vivo studies in rats [46]. In liver microsomes isolated out of sheep and cattle, the conversion of formononetin to DAI was found to be very low. Further conversion to EQ could not be induced by these microsomes [60]. Studies with microsomes of other farm animals are currently unavailable.

The oxidative metabolites of ISF formed in the liver may undergo enterohepatic circulation and therefore, further metabolic conversion by the gut microbiota could be initiated as shown for EQ [61]. Due to their pyrogallol and catechol structure, the oxidative metabolites show a lower stability than their precursors and are therefore difficult to quantify. For humans, it was estimated that less than 10% of the total ISF content is present as oxidative metabolites in urine [62], whereas bacterial metabolites are of greater importance [63].

### 2.3. Phase II Metabolism

In the further process, ISF are converted to their glucuronides and sulfates in the intestine and liver by means of enzymatic conversion. SULT1A1, among other sulfotransferase (SULT) isoenzymes, is responsible for sulfation, and isoenzymes of uridine diphosphate (UDP)-glucuronosyltransferase (UGT) accomplish the conjugation with glucuronic acid [53]. These conjugation reactions are considered to be the main detoxification pathway for ISF. Cheetahs, for example, which cannot conjugate ISF, showed symptoms of liver damage and infertility after a soy-containing diet [64].

Conjugation occurs mainly at the hydroxy group at C-7 [65,66,67]. Predominantly monoglucuronides, but also diglucuronides, mono- and disulfates, and sulfoglucuronides were reported [53]. However, the pattern of phase II metabolites differed not only between species, but also between genders. In humans, the main conjugate of phase II metabolism for DAI was the 7-glucuronide-4′-sulfate. For GEN, the metabolites 7-glucuronide-4′-sulfate as well as the 7,4′-diglucuronide predominate. However, differences between sex were not observed in humans. Rats, on the other hand, showed a divergent phase II metabolite profile between genders. Female rats displayed preferred production of 7-glucuronides for both GEN and DAI. The male counterpart exhibited primarily the production of 7,4′-disulfate and 7-glucuronide-4′-sulfate [68]. A possible explanation for the prioritizing formation of sulfates in male rats can be explained by elevated SULT1A1 mRNA levels [69].

Conjugation with sulfates and glucuronic acid occurred predominantly in the liver, but may also involve the gastrointestinal tract [70]. The gastrointestinal epithelium represents an important location for detoxification of phytoestrogens in ruminants. However, there are also differences between species. The conjugation activity of the ISF formononetin, DAI and EQ was up to 20 times higher in sheep compared to cows in almost all parts of the gastrointestinal tract. Among the three ISF mentioned, EQ showed the highest conjugative activity for both species [71].

### 2.4. Distribution

Following absorption and metabolization in both the intestine and liver, ISF are transferred to various body fluids and tissues. These include plasma, urine, feces, and milk on the one hand and kidney, liver, ovary, uterus to name a few on the other hand [53,72,73,74]. In addition, there is evidence that ISF can cross the blood-brain barrier as well as the placenta [75,76]. Depending on the animal species, ISF and their metabolites are present more or less conjugated in plasma and urine. Although in pigs the proportion of aglycones in these fluids is usually less than 5%, the proportion of non-conjugated ISF in urine may be up to 50% in rats and up to 90% in monkeys [53]. ISF are also found in tissues in different forms depending on the animal species. For example, in rats the ISF are predominantly present as aglycones, whereas in sheep the vast majority of ISF were detected in conjugated form [73,77].

### 2.5. Inter-Individual Variability

Several different factors may influence biokinetics and bioavailability of ISF such as intestinal microflora, age, composition of feed, and duration of soy consumption. Contradictory data are available on the concentration of EQ in the blood of pigs. Although one study did not detect EQ in the serum of piglets (age 30 days) [53], another study detected EQ in serum of pigs (age 183 days) [78]. This observation might be explained by the different age of the investigated animals. This phenomenon was also observed in rats. Although older rats can produce EQ, no EQ was detected in 3 months old rats [79]. This is attributed to the presence or absence of certain bacterial species that establish itself in the gut in the course of a lifetime [79].

The composition of the feed also seems to have an influence on the metabolism and bioavailability of ISF. It appears that a higher proportion of carbohydrates in the diet and a reduction in fat favors EQ production [80]. Higher levels of carbohydrates may stimulate fermentation in vitro and contribute to increased EQ formation [81]. Likewise, an increase in bioavailability may occur when the feed consists to a minor percentage of oligo-fructose as has been the case in rats for DAI and GEN [82]. In addition, genetic polymorphisms of xenobiotic-metabolizing enzymes such as CYP and UGT may affect bioavailability and metabolism. The genotypes (Val/Leu) and (Leu/Leu) of the gene *CYP1B1* are suspected to contribute to an increased risk of breast cancer at a low energy-adjusted daily soy ISF intake [83]. Likewise, the gene polymorphism UGT1a1*28, which is responsible for the conjugation of xenobiotics, has an influence on the metabolic pattern. The presence of this polymorphism in individuals leads to increased excretion of GLY-, DAI-, and GEN-sulfates, causing a decreased production and urinary excretion of glucuronides [84].

### 2.6. In Vivo

#### 2.6.1. Ruminants

**Figure 3:**
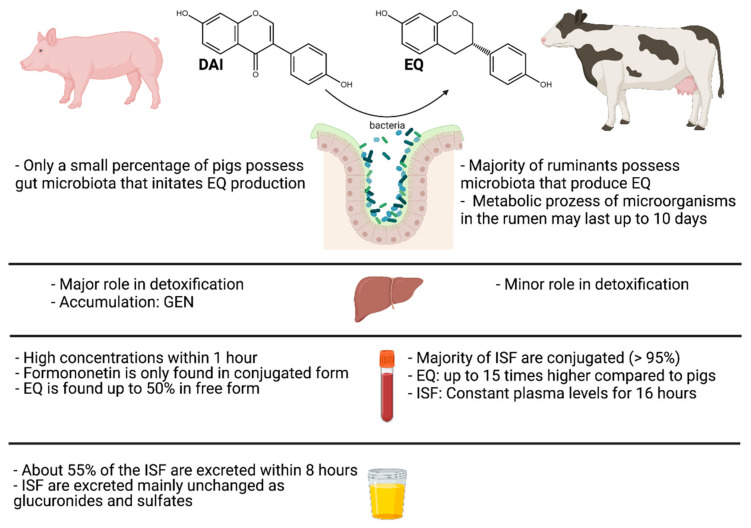
Comparison of the metabolic pattern of ISF between pigs (**left**) and ruminants (**right**). ISF = isoflavone(s); DAI = daidzein, EQ = equol; GEN = genistein. To the best of our knowledge no data are available concerning the excretion of ISF in ruminants. Figure created with BioRender.com.

Compared to monogastric animals, the ruminant’s stomach works in a completely different way. Therefore, it is not surprising that also the metabolization of phytoestrogens is different (Figure 3). The rumen is the primary location of the deglycosylation of the ISF glucosides mainly present in the plants, and other transformation processes of the aglycones [48]. Biochanin A is metabolized by demethylation to GEN and further by ring cleavage to para-ethylphenol and organic acids [85]. Formononetin is predominantly demethylated to DAI [85]. EQ is formed from DAI by hydrogenation and ring cleavage. Contrary to humans and pigs, ruminants are principally EQ producers [85]. The metabolites formed from biochanin A and GEN are estrogen-inactive substances. Metabolism of formononetin, however, leads to formation of the more estrogenic metabolite EQ [86]. The metabolic processes catalyzed by microorganisms in the rumen may last six to ten days after ingestion [87]. In cows, an ISF-rich diet can play an essential role in their metabolism. One study investigated the degradation of dietary ISF in rumen fluid from cows fed with either a hay diet or a concentrate-rich diet, both including 40% soybean extract. The results showed faster metabolism of both DAI and GEN under concentrate-rich conditions and an overall higher production of EQ under the hay diet conditions [88]. No significant differences were observed in GLY degradation between the two diet conditions. However, using higher amounts of soybean extracts up to 75 mg per 40 mL rumen fluid resulted in a decrease in EQ production, most likely because of the inhibitory effects of GEN on the rumen microflora [88]. This decrease is in agreement with a previous study [50]. Only a minimal percentage of hydrolyzed phytoestrogens is absorbed directly from the rumen into the bloodstream [85]. The majority is first subject to further conjugation, predominantly with glucuronic acid [85]. This already takes place in the gastrointestinal epithelium and only a tiny percentage is conjugated in the liver [85]. This fact suggests that in ruminants the liver plays a minor role as an organ for ISF detoxification [85].

Feeding trials performed in dairy cattle and ewes revealed that the absorption and distribution of ISF differs within these two species. Formononetin (530 mg/kg feed) and DAI (12.6 mg/kg feed) were absorbed very rapidly in dairy cattle, reaching concentrations of about 90 and 50 µg/L in blood plasma, respectively [51]. The concentration of these two ISF was three times higher within the first hour compared to the concentration detected in sheep when fed with the same ISF concentration [51]. The EQ concentration in dairy cows was maintained at a constant level of about 180 µg per 100 mL plasma over a 16-h period. Initially, sheep showed a lower total (free and conjugated) plasma EQ concentration, which increased after three hours to the same level as in cows. However, after 16 h, the EQ concentration was half as high as in dairy cows, indicating a faster metabolism of ISF in sheep [51,85]. The amount of unconjugated ISF in the blood was ≤5% for either species, whereby the concentration of free EQ was 10-fold higher in dairy cows compared to ewes at all time points. Nevertheless, sheep are described to be more sensitive to EQ [85]. One of the most plausible explanations is that the estrogen receptors in the uterus are expressed 2–4 times more in sheep than in cows [89,90]. Considering that the relative estrogenic potency of EQ is 0.061% of 17-β-estradiol [91] and that free EQ in sheep can reach concentrations of 20 ng per 100 mL, EQ could reach a 100-fold higher estrogenic potency than 17-β-estradiol during estrus [92].

#### 2.6.2. Pigs

The metabolic profile of ISF in pigs was investigated in a feeding trial with diets containing 20% red clover with previously determined levels of ISF, corresponding to an average daily intake of 97 mg GEN, 88 mg DAI, 866 mg formononetin and 378 mg biochanin A [85]. Using a permanent vein catheter, the concentrations of the above-mentioned ISF could be determined at different time points after feed intake. Within one hour the total (free and conjugated) maximum level of formononetin (100–120 µg/100 mL plasma) was 10 times higher than the level detected in bovine plasma that was also investigated in this study. This observation that formononetin can be detected very rapidly in blood plasma suggests that it is already absorbed to a large extent in the stomach. DAI (5–7 µg/100 mL plasma) and EQ (12–25 µg/100 mL plasma) likewise exhibited the maximum level detected within one hour of feed ingestion, although at 5–15 times lower concentrations compared to formononetin [85]. Other feeding trials have shown that depending on the feed composition, different ISF are primarily detectable in plasma. Measurements after feeding of a soybean meal (SBM) on the one hand and soy protein concentrate (SPC) on the other hand showed that after hydrolytic cleavage of phase II metabolites such as glucuronides and sulfates of ISF, DAI and GEN were detected in higher amounts in the plasma of SBM fed animals [93].

Formononetin was almost exclusively found in the conjugated form, whereas EQ was found up to 50% in free form in plasma [85]. However, the total amount of EQ in pigs is up to 15 times lower compared to other species such as ruminants [85]. The different ability to produce EQ between species is largely due to the different composition of the gut microbiota. Thus, it appears that pigs in comparison to ruminants possess only a small percentage of the gut microbiota that initiates the conversion to EQ [53]. However, the fact that EQ is detectable in blood and tissues of pigs strongly suggests that pigs are also EQ producers [78,93]. Although Lundh et al. found EQ as a major metabolite of soybean and red clover ISF in pig plasma, Gu et al. detected no EQ in pig serum [53,85]. The difference between these observations might be due to the age difference. In young piglets the intestinal microflora might not be developed sufficiently to produce EQ.

The major metabolites recovered in mammary tissue of pigs were DAI and EQ [78]. These two ISF exhibit estrogenic activity, and both may accumulate in tissues of the reproductive tract and thus strongly affect the reproductive system in pigs [78]. In contrast to DAI and EQ, GEN was mainly detected in the liver of pigs [78]. After absorption, ISF are converted to their glucuronides and sulfates in the intestine and liver by enzymatic conversion [53]. Sulfate conjugates are thought to have a higher estrogenic potential compared to glucuronides [94,95].

About 55% of the ISF were excreted via urine within the first 8 h after feed intake [85]. In the case of formononetin, 72% was excreted via urine without metabolic changes [85]. GEN was also excreted mainly unchanged via urine and only to a minor percentage the metabolite dihydro-GEN was detected [53]. In addition to DAI as the main component of urinary excretion, the metabolites *O*-demethyl-angolensin (ODMA), EQ, and DHD were determined [53].

#### 2.6.3. Poultry

Several in vitro and in vivo studies on the effects of ISF have been conducted in poultry, especially chickens. Soybean ISF constitute a large part of their diet, and these phytoestrogens transfer to and accumulate in the tissues and eggs of hens [96,97].

In 2001, an experiment conducted at the Tokyo University of Agriculture revealed the transfer of soy ISF to the plasma and egg yolk of laying hens. A group of 5 laying hens was fed a high dietary concentration of soy ISF extracted from soybean hypocotyls over an 18-day period. The concentration of ISF in the ISF-enriched diet was at least 3 times higher than the normal diet, containing for the enriched diet 353 mg/kg, 26.2 mg/kg and 476.9 mg/kg of DAI, GLY, and GEN, respectively. Analytical data showed a sharp increase in the concentrations of total ISF in plasma and egg yolk until day 12 of feeding with values of 3.2 nmol/L in plasma and 65.3 µg/100 g in egg yolk [96]. Another study reported the transformation of DAI to EQ which then accumulated in the egg yolk of laying hens. The results showed that although most of the ISF (DAI, GLY, GEN) were present in blood and yolk, the concentration of EQ was much higher than the concentrations of other ISF, especially in the egg yolk [97].

## 3. Effects of ISF

### 3.1. Ruminants

Several studies have been conducted in ruminants such as cows and sheep reporting both positive and negative effects on the health of these animals. Several studies revealed the excretion of ingested ISF in ruminant’s milk and tissues, as well as increased growth and reduced fertility upon ISF exposure [21,22,23,24].

A study investigated the effects of ISF-enriched feed on the carry-over of ISF to milk and on the rumen microbiota in lactating Czech Fleckvieh x Holstein cows. The experimental group received a basal diet supplemented with 40% more soybean ISF extract (16,006 mg/day) compared to the control group (8401 mg/day). As a result, the concentration of EQ in milk was nearly 2.5 times higher in the experimental group. Additionally, the experimental cows had a reduction in microbial richness compared to control cows [22]. Interestingly, several publications report a high concentration of EQ in bovine milk. A study in Denmark reported a very high EQ concentration of 230 µg/L in organic milk [98]. An even higher EQ concentration of 293 µg/L was detected in the milk of Australian cows that received a high clover diet [99]. Similar to soybean, red clover (*Trifolium pretense* L.) contains phytoestrogens with the prominent ones being formononetin and biochanin A [100]. It plays a vital role in agricultural processes and has a high crude protein content and fiber, making it an important and feasible option for feeding ruminants. Dairy cows fed with red clover silage have increased milk production as well as higher intake of nutrients compared to animals fed green silage [23,24,101,102]. Similar to the ISF measurements in Australian and Danish bovine milk, two studies performed in France found EQ concentrations of up to 191 µg/L and 1120 µg/L in cow and goat milk, respectively [103,104]. Though DAI and GEN were also present in milk, their concentrations were lower compared to EQ concentration [99,103,104]. Furthermore, an EQ concentration of 364 µg/L in cows fed with red and white clover silage was also reported [9]. These results are in line with another study where cows were fed with a similar diet [105,106].

The concentration of EQ has been studied not only in milk, but also in meat and tissues [73,107]. In ewes fed a diet of red clover, EQ and DAI found as glucuronides reached high concentrations in plasma. However, these compounds were not equally distributed in different tissues with the highest amount found in the kidney [73]. Lambs fed with red clover pastures showed higher weight gain and fiber intake compared to those receiving a ISF free diet [19,20,108]. These observations were in line with another study where red clover-fed lambs had gained more weight than those fed a white clover pasture [109]. Another supporting trial also reported faster growth rates of lambs fed with red clover silage [110].

Though phytoestrogens can have a positive effect on milk production of cattle, they can also lead to adverse effects on the reproductive system of both cows and sheep. Several studies have been carried out to test the effects of ISF on the reproduction rate and reproductive tract of the animals, and varying results have been observed in both species [87]. A series of pathological examinations in animals fed mainly with red clover showed signs of cervicitis and hyperplasia in the endometrium of sheep [87]. Chronic infertility was observed in sheep after continuous exposure to ISF, reflected by symptoms that included irreversible changes in estrogen-sensitive organs and completely masculinized ewes [87]. In cattle, only temporary infertility has been observed so far due to ISF exposure. This is reflected by a reduced conception and ovulation rate [87]. In addition, other symptoms such as irregular estrus cycles, vulvar swelling, cyst formation and behavioral abnormalities such as absence of estrus and nymphomania were reported [87]. After discontinuation of ISF exposure, symptoms decreased over a few days to weeks and disappeared with time [87]. Cattle are less sensitive to exposure to ISF compared to other ruminants and this is suspected to be due to differences in estrogen receptors and tremendous weight differences [51]. Formononetin and DAI, which are converted in the rumen into the highly estrogenic EQ, are mainly responsible for infertility [111]. Only three out of five cows fed a soybean-rich diet became pregnant after successful insemination, compared to four out of five cows in the control group [112]. Furthermore, in the same study, cows that received a soybean-rich diet showed elevated levels of prostaglandin F_2α_ in the blood that were positively correlated with EQ concentrations. The ISF GEN and biochanin A have also been associated with infertility [113]. In one case study, a red clover silage diet caused vaginal discharge and irregular estrous cycles leading to higher miscarriages and premature conception rates in cows [114]. These changes were reversible when feeding of red clover was discontinued [114]. In agreement with these observations, other studies reported the onset of vaginal prolapses, infertility, increased udder size and growth of ovarian cysts in cows fed with red or subterranean clover [115]. Furthermore, cows that received a soy-containing diet showed a lower pregnancy rate and needed a higher number of artificial inseminations than cows that received soy-free diet [112]. In contrast, one study described increased fertility of heifers fed red clover silage [116]. In this study, the heifers were fed either red clover or grass silage prior to and during the insemination period. Cows that received the red clover silage diet showed a significantly higher pregnancy rate to first service, with an increase of 33% [116].

Infertility of sheep fed with a clover diet gave rise to the so-called “clover disease” [28]. The disease can be classified according to certain clinical conditions such as infertility in ewes that goes hand in hand with changes in the endometrium, prolapse of uterus in unmated ewes and the death of lambs during delivery due to failure of the cervix to dilate properly, mammary development and lactation in unmated ewes and wethers (castrated male sheep), blockage of urethra and death in wethers [21]. Furthermore, sheep that were fed a clover-rich diet showed a loss and or reduction in reproduction [117]. The effects of ISF can cause two different types of infertility problems in sheep, either homeostatic reproduction was recovered after the absence of clover diet or infertility worsening with continued clover-feeding [21]. Indefinite and irreversible infertility in ewes is characterized by a decrease in pregnancy rates, secretory function changes of the cervix and loss of the mucous membrane [21,118,119]. Late pregnant ewes that received clover silage from two months prepartum until estrus induction showed a higher interval to estrus, shorter estrus duration and tended to show a decreased litter size compared to ewes that received maize silage [120]. Red clover silage with a high phytoestrogen content fed to nulliparous ewes before, during and after the breeding season did not reduce fecundity compared to a control group that received timothy/meadow fescue grass silage. However, the volume of fetal fluids increased in ewes that received red clover silage, which could increase the risk of vaginal prolapse before the term [121].

In Sweden, a study investigating the effects of dietary phytoestrogens on plasma testosterone and triiodothyronine (T3) levels in male goats was conducted [122]. From 3 months of age to 6 months of age, the goats received either a basal diet or a diet rich in phytoestrogens. In the first 7 weeks, no significant difference in the concentration of plasma testosterone was observed between the two groups. By the fifth month, the goats fed the ISF-rich diet showed significantly higher testosterone concentrations compared to the control animals. Additionally, the concentration of free T3 was also higher in animals that received ISF-rich feed. These findings suggest that ISF can promote testosterone synthesis during the puberty stage of male goats by increasing secretion of T3 [122].

Concluding, in ruminants, different effects of ISF were reported depending on the animal species and the study design. Cows displayed an increase in metabolism and milk production when they received ISF-rich feed. Animals fed clover silage especially showed increased milk production and nutrient intake [23,88,101,102]. A high concentration of EQ, DAI, and GEN was found in the milk of cows, as well as EQ and DAI in the plasma, meat, and tissue of sheep [73,105,107,123]. Similar to cows, lambs fed with red clover pastures (ISF-rich diet) reported higher weight gain and fiber intake, contributing to a faster growth rate of these animals [109]. Unlike poultry, ISF led to an adverse effect on the reproductive system of ruminants, where they caused either a temporary or permanent form of infertility in sheep. The effects of ISF on the reproduction and fertility rate of cows was not as profound, with a few reported miscarriages and infertility which was reversible [51,87]. Additionally, in male goats, ISF promoted the onset of puberty by increasing testosterone synthesis [122]. Since soy is one of the most widely used protein sources, an adverse effect on the reproductive system due to the high content of ISF cannot be excluded. In fact, an influence of soy-rich diets on female reproductivity in cows can be observed.

### 3.2. Pigs

Phytoestrogens may affect the growth, meat quality, and immune response system of pigs and are therefore of great interest for the swine agriculture industry. There are several studies on the effect of soybean meal on the growth, intestinal morphology, and antioxidative properties in pigs and piglets. In one experiment, groups of piglets were fed different types of feed, i.e., corn-soybean meal (C-SBM), corn-soy protein concentrate (low ISF C-SPC) or C-SPC enriched with the same ISF concentration as in C-SBM over a period of 72 days [25]. Piglets fed the C-SBM and C-SPC + ISF diets showed higher body weight and greater villus height on day 72 than piglets that received C-SPC feed. In comparison to the C-SPC diet, the pigs fed with dietary C-SPC + ISF displayed an increase in plasma superoxide dismutase (SOD) activity on days 28 and 42. This was also followed by a reduction in plasma malondialdehyde content on day 42. In conclusion, the feeding of soybean ISF over a long period of time improved growth performance and antioxidative activity, as well as protection of the intestinal morphology [25]. Lipopolysaccharide (LPS)-induced retardation in growth performance, diarrhea, and high plasma concentrations of endotoxins and malondialdehyde have been reversed by supplementation with ISF [124]. Furthermore, LPS challenge significantly increased the abundance of p-p38 and TRL4 proteins in the jejunal mucosa of piglets which are thought to be involved in intestinal damage. The addition of ISF resulted in a decrease of these proteins [124]. A case study determining the effects of dietary soy ISF on growth, carcass traits and meat quality in nearly fully grown pigs was performed [125]. Pigs were allocated to three different diets: (1) C-SBM; (2) C-SPC or (3) C-SPC + ISF. Pigs fed the C-SPC + ISF diet showed an increase in carcass length, percentage ham lean and thaw loss, combined with an overall reduction in total ham fat [125]. Pregnant sows that received dietary DAI and GLY from day 85 of gestation littered piglets with higher birth weight and average daily gain [18,126]. However, the effect of ISF on growth performance is contradictory in the literature. In a study by Xiao and coworkers, piglets that received a dietary DAI level of 400 mg/kg showed lower average daily weight gain than the control group that did not receive DAI [15]. The discrepancy between the observed effects of ISF on growth performance may be due to differences in age of the animals, duration of the study or the kind and dose of ISF. Furthermore, such high DAI concentration had a trend to cause mild damage of the kidneys, livers and spleens of the weanling pigs after 70 days [15].

Several studies have reported beneficial effects of ISF in pigs challenged with viral infections. One study investigated the effects of dietary SBM infused with ISF on the growth performance and immune response of pigs inoculated and infected with porcine reproductive and respiratory syndrome virus (PRRSV). The effect of ISF enhanced immune response by reduced concentrations of serum viral load and improved growth [127]. This agreed with another study about the effects of dietary soy ISF on the response of weanling pigs to PRRSV. The ISF supplemented to the pigs reduced PRRSV-induced circulating neutrophils and improved the cytotoxic-to-helper T-cell ratios [128]. The findings suggest that ISF contribute to activating the adaptive immune system and in clearance of viral infections [128]. Furthermore, the effect of dietary soy GEN on pig growth and viral replication was also investigated. Increasing concentrations of dietary GEN were associated with a decrease in serum content of the PRRS virus and a quadratic increase in daily feed intake [129]. Furthermore, a study by Smith and coworkers found that dietary ISF reduced the mortality of pigs challenged with PRRSV [130].

One study investigated the effects of a high DAI dose (640 mg/kg feed) on the redox system in tissues of finishing pigs over a period of 64 days [16]. On the one hand, the supplemented high dosage increased SOD activity and total antioxidant capacity in the longissimus muscle of the animals, and it reduced activity of NADPH oxidase-2 and cyclooxygenase-2, enzymes involved in the production of reactive oxygen species. However, on the other hand, it also induced pro-oxidant changes in back and abdominal fat, liver, and plasma tissues by promoting the expression of reactive oxygen species producing enzymes. To conclude, finishing pigs fed with high doses of DAI show improved redox activity in muscle and, in contrast, increased oxidation levels in liver and fat tissues [16]. Similar to its precursor DAI, the metabolite EQ also displays antioxidant effects in piglets. A case study reported that lactulose increased EQ production and improved liver antioxidant status in barrows supplemented with DAI [131]. After 20 days, EQ concentrations were significantly elevated in both urine and fecal samples. Additionally, the activity of the enzymes SOD and copper-zinc SOD increased in the livers of barrows, thus improving the overall antioxidant characteristics of the animals [131].

The effects of ISF on the reproductive system of pigs have also been studied, though not as much as in sheep and cattle. A study from China investigated the effects of soybean ISF on male reproductive parameters using Chinese mini-pig boars as a model [132]. Dietary concentrations of soybean ISF of 0 to 500 mg/kg were administered to the pigs for 60 days. An ISF concentration of 250 mg/kg increased the testis index, fructose content in testicular tissue, alpha-glycosidase content in testicular tissue, as well as viable germ cells and level of apoptosis regulating Bcl-2 protein in testicular tissue. Pigs fed 500 mg/kg of soybean ISF exhibited a significant decrease in testis and epididymis indexes and serum testosterone levels, as well as an increase in numbers of early and late apoptotic germ cells and level of pro-apoptotic BAX proteins in the testis. In conclusion, low to moderate consumption of soybean ISF did not affect reproductive parameters in the mini-pig board, whereas higher concentrations negatively affected male reproductive health [132].

Similar to the Chinese mini-pig boars, another study also investigated the effects of soybean ISF on the onset of puberty, serum hormone concentration, and gene expression in hypothalamus, pituitary gland and ovary of female Bama miniature pigs [133]. The pigs received dietary soybean ISF doses between 0 and 1250 mg/kg. Compared to the control group, the pigs fed 1250 mg/kg soybean ISF exhibited a significant delay in the onset of puberty. At 4 months of age, a reduction in serum concentration was observed for gonadotrophin releasing hormone and luteinizing hormone, whereas the serum concentration of follicle-stimulating hormone increased. The soybean ISF-supplemented pigs also showed a reduction in ovary of steroidogenic acute regulatory protein and KiSS-1 metastasis-suppressor. To conclude, soybean ISF adversely affected the reproduction system in female pigs by delaying the onset of puberty due to the decreased expression in reproduction-regulating genes [133].

Feeding red clover silage to pigs induced signs of hyperestrogenism such as fertility problems [85]. Further feeding trials of 10- to 24-week-old piglets with soy showed that the soy-containing diet induced estrogenic effects in the piglets [134]. Again, symptoms of hyperestrogenism such as swelling of the mammary gland and vulva, enlargement of the uterus, and pathological changes of ovaries were observed. These data also suggest that with continuous feeding of soy, the vulva size increased with each week. This observation implies that there is an enhanced estrogenic effect over time due to cumulative effects [134].

Ovariectomized gilts are often used as a model to study estrogenic properties of different substances. Because they have a deficit in estrogens, the effect of mildly estrogenic substances can be determined. Therefore, Ford and coworkers [135] studied the effect of GEN in ovariectomized gilts. The gilts received daily intramuscular injections of 50–400 mg GEN. Doses of ≥200 mg caused a significant increase in cervix and uterine mass.

DAI exposure of pregnant sows caused swelling and reddening of the vulva in neonatal piglets [136]. Compared to the control group, the male newborn piglets whose mothers were fed with DAI had a significantly higher birth weight. No other influences on the piglets were observed. However, the addition of GEN and DAI to the feed of lactating sows caused estrogenic effects in the piglets, which are most susceptible to exogenous endocrine disruptive chemicals (EDC) at this stage of their lives. It can be concluded that ISF exposure through breast milk led to an exposure of the estrogenic substances to the piglets causing hyperestrogenism in female piglets [136].

In conclusion, ISF were shown to exert both positive and negative effects in pigs. Soybean meal improved the growth performance and antioxidative activity in pigs [15,124,125,126]. In PRRSV-challenged pigs, ISF improved immune functions and reduced mortality [129,131]. However, phytoestrogens also showed negative effects. ISF caused hyperestrogenism in female piglets [85,134]. Similar to what was observed in sheep, high consumption of soybean ISF negatively affected male reproduction [132]. Furthermore, high doses of ISF were reported to have toxic effects including increased oxidation levels in liver and fat tissues [16] and soybean meal fed pigs showed reduced total ham fat [124].

### 3.3. Poultry

Chicken that were fed with a soy ISF-rich diet (starting at 1000 mg ISF/kg feed) showed significantly higher plasma levels of estradiol compared to chicken on a basal diet. The same chicken were also much healthier as shown by improved egg and white egg weight over the course of the experimental period of 3 months [17]. The improvement in egg weight is in agreement with increased egg production and quality, as reported in previous studies [96,137]. Studies investigating the effects of soy ISF (DAI, GEN, GLY) on the laying performance of Xueshan breeder hens found that an ISF diet increased yolk color and serum total antioxidant capacity and immune levels [138,139,140]. Furthermore, ISF caused an increase in hatching rate potential of 36-weeks old hens [138]. However, ISF caused a reduction in average egg weight in 52-weeks old hens and a decrease in egg rate in 44-weeks old hens [138]. Dietary administration of DAI to laying hens at doses of 10, 100 or 200 mg/kg for 12 weeks increased hatchability [139]. Furthermore, dietary DAI doses of 10, 20 and 30 mg/kg showed a positive effect on egg weight and fertility in Zhedong white geese [141].

Ni and coworkers investigated the effect of DAI on the egg-laying performance in Shaoxing duck breeders [137]. The following criteria were measured: egg-laying rate, egg composition, feed conversion ratio, hatchability characteristics of eggs and body weight, ovary and oviduct weight, and changes in serum concentrations. Doses of 3 mg/kg and 5 mg/kg DAI administered to ducks over 35 days resulted in an increase in the egg-laying rate, mean egg weight, and feed conversion ratio. However, a negative effect on fertility and hatchability responses was observed. Extending the feeding period to 63 days caused a 7.7% increase in the egg-laying rate, as well as higher body and oviduct weight. Additionally, the yolk and albumen ratio were reduced. This suggested that DAI has ambiguous effects on the laying performance of the ducks depending on the physiological conditions and DAI dose [142]. The impact on the laying rate seems to depend on the age of poultry. In 7-months old female quails, supplementation of 6 mg/kg DAI led to a decrease in laying rate, whereas the same amount of DAI led to an increase in laying rate in 12 months old quails [13].

Different effects have been observed in Japanese quails. In one study, ISF administered to female quails via the diet did not affect growth, feed intake or weight of oviduct [14]. Likewise, ISF did not affect growth or feed intake in male quails in the same study. However, the same male quails exhibited a 40% reduction in photoperiod-induced testis development suggesting ISF negatively affect the reproduction system of male quails [14]. It was reported that the supplementation of GEN in Japanese quail suppressed spontaneous oviduct tumorigenesis. The results indicated that GEN supplementation significantly reduced the occurrence and size of spontaneously occurring leiomyoma of the oviduct in the quail [143].

In a study in broiler chickens very high dietary concentrations of soy ISF (starting at 693 mg/kg) decreased the growth rate [144]. In contrast, the administration of lower ISF concentrations (10 and 20 mg/kg) increased the weight and feed intake of male broilers compared to the control group [145]. Following, one study could show that supplementation of GEN (400 mg/kg) fed for 8 weeks significantly improved the reproductive activity and bone status of laying broiler breeder hens. The GEN-rich diet induced an increase in the levels of vitellogenin, progesterone, and follicle-stimulating hormone in the serum [146]. In addition, the levels of malonaldehyde in the follicle and egg yolk of hens decreased, while calcium and phosphorus levels increased in the tibia, which explains the improved strength of tibia bone [146].

Antioxidant effects of ISF have also been observed in poultry. One study highlighted the positive benefits of an ISF-rich diet on broiler chickens suffering from the infectious bursal disease virus (IBDV). Dietary ISF improved the overall health and condition of infected chickens. This effect was attributed to the decreased expression of viral protein 5 mRNA, a protein produced in response to IBDV to drive apoptosis [147,148]. ISF also decreased the onset of bursa lesions and additionally had a high antioxidant capacity [147]. Further antioxidative properties of ISF in male broiler are described by the consumption of 40 or 80 mg ISF per kg bodyweight, which leads to an increased antioxidant capability and superoxide dismutase activity in plasma [145].

To conclude, in poultry, high ISF concentrations in the diet increased the rate of egg production and hatching rate due to increased estrogen activity and weight in some studies [138,149]. However, ISF decreased average egg weight in 52-weeks old laying hens and egg rate in 44-weeks old laying hens [138]. High ISF concentrations were linked to improved antioxidant effects and immune system as well as a reduction in blood cholesterol levels, improving overall health, and such rich diets decreased the onset of bursa lesions and viral mRNA in chickens infected with infectious bursal disease virus (IBDV) [140,147,150]. Additionally, the ISF GEN affected the reproductive system and bone status of hens and ducks, by capacitating their performance and enhanced strength of their tibia bone [146]. Several negative effects of ISF were observed in quails. Dietary ISF caused a decrease in the laying rate of 7-months old female quails [13] and negatively affected the development of the reproductive tract in males [14].

### 3.4. Fish

Studies about the effects and the metabolic fate of ISF on aquatic species are scarce. However, one research team showed that the supplementation of soy ISF to the diet of the marine fish “golden pompano” caused an increased growth rate [151]. In contrast, higher levels of ISF supplementation in diets of some other fish species such as Japanese flounder, Atlantic salmon fry and yellow perch led to a significant reduction in the weight of the animals [152,153,154]. Therefore, a general conclusion is not possible and in other marine species, such as striped bass fingerlings, no effect of ISF on the growth was observed [155]. These differences in the effect of ISF-supplemented diets may be due to different ISF pattern or might be species dependent. ISF supplementation is known to alter the digestive process by inducing a reduction in maltase activity, which is a key enzyme in the digestion of carbohydrates in fish [154]. In addition, a tendency towards skeletal malformation was observed in ISF-fed Atlantic salmon presumably related to the reduction of thyroid peroxidase [154]. Thyroid peroxidase is mainly involved in the synthesis of thyroid hormone (TH), which in fish plays a crucial role in the development of the musculoskeletal system [156]. Another factor that may contribute to the impaired growth of salmon fry is the depletion of glycogen in hepatocytes induced by ISF-containing diets [154].

Studies indicate that consumption of ISF leads to a decrease in whole body crude lipid content in juvenile Japanese flounder [152]. It was suggested that this decrease is due to an effect of ISF on transcription factors which modulate the expression of genes involved in lipogenesis or lipolysis [157]. Considering that there is little data on the metabolism of ISF in fish, it is also inconclusive whether fish can produce EQ or not. For example, no EQ was detected in the bile of rainbow trout fed with DAI (up to 49 mg/kg feed) [158], whereas EQ could be detected in the tissue of sturgeons (EQ intake up to 432.3 mg) [159]. However, it cannot be concluded from these results that sturgeons are in fact EQ producers as EQ was also present in feed. Additionally, the ISF profile detected in the serum indicates that sturgeons did not produce EQ in this experiment [159].

The metabolism of ISF in sturgeons seems to be delayed, as high concentrations were found in the liver (up to 3.5 mg ISF per kg liver), indicating an accumulation [159]. This accumulation can cause a chronic estrogenic effect in hepatocytes, which is reflected by increased vitellogenin synthesis observed both in vivo and in vitro [160,161]. In Siberian sturgeons, intraperitoneal administration of GEN, EQ, and biochanin A increased vitellogenin levels in blood, thus exerting an estrogenic effect [162]. Therefore, particular attention should be drawn to GEN because of its high occurrence in fish feed and accumulation in tissues, which can result in particularly high estrogenic effects [162]. In rainbow trout, GEN, DAI, and GLY can inhibit the metabolism of E2 in the kidney and liver. Inhibition of E2-metabolizing enzymes may result in increased bioavailability of E2 in peripheral target tissues. This could be another potential mechanism of how ISF induce estrogenic effects [163].

ISF had a positive effect on immune parameters in golden pompano. Feeding 40 mg/kg ISF resulted in a significant increase in C3 protein, which is part of the humoral immune response and plays a central role in the lysis of pathogenic cells and bacteria [151]. Moreover, there was an increase in plasma lysozyme activity due to ISF. Lysozyme activity is an important index of innate immunity, which plays a more important role in fish than in mammals. Numerous other immune parameters and health indicators such as increased activity of respiratory bursts, decrease of glutamic-pyruvic transaminase and oxalacetic transaminase and increase of HSP70 were induced by ISF supplementation [151].

## 4. Occurrence

### 4.1. Literature

As pointed out in the introduction, significant concentrations of ISF are mainly found in plants of the *Fabaceae* family [164]. However, ISF content as well as the overall composition of soybeans and red clover are subject to wide variations depending on cultivar, season, and further processing [165].

In addition to contamination with ISF, contamination of feed and food with zearalenone (ZEN) and its metabolites may occur at different stages of the feed supply chain. ZEN-producing *Fusarium* species grow well in humid weather on the fruit or stalk of grain and their growth is accelerated by improper storage. Contamination with ZEN and its metabolites is common in commodities such as wheat, corn, rice, barley, soybean. [166,167,168].

The occurrence of ISF and the incidence of ZEN in a diverse range of feedstuffs analyzed over the last 10 years is provided in Table 1. Although there are numerous studies regarding the occurrence of ZEN and its metabolites in feed over the last decade, there is only scarce data on the incidence of ISF. However, in all samples the detected average concentrations of ISF are in the medium to high mg/kg range. In silage samples that are composed of clover, grass, and cocksfoot, the prevalent ISF are formononetin and biochanin A and this in accordance with previous reports [100,169].

The situation is different for soybean meal and cow feed samples, where the glycosides genistin and daidzin are the predominate ISF. Cow feed samples from Thailand reached maximum concentrations of 57 and 42 mg/kg feed for genistin and daidzin, respectively [40]. The concentrations of these two ISF in soybean meal feed were even higher, reaching 1274 and 785 mg/kg feed for genistin and daidzin, respectively. No cases were reported where the aglycon showed higher concentrations than its respective glucoside [40]. Compared to the samples from the US, soybean feed samples from Brazil and Argentina had higher concentrations of ISF, which could be due to environmental factors that lead to higher concentrations of ISF in soybean meal [72]. The prevalence of ZEN was in most of the samples very high. Some samples also exceeded the EU guidance values for acceptable ZEN concentrations in pig feed [167] (EU guidance values for: piglets and gilts 0.1 mg/kg; sows and fattening pigs 0.25 mg/kg; calves and dairy cattle 500 µg/kg). Feed samples were found that exceeded the guidance value for piglets and gilts in every region of the world. For samples from East Asia, 27.3% of the analyzed samples exceeded the guidance value for piglets and gilts [167].

Among the few studies investigating the co-occurrence of ISF and ZEN or its metabolites in feed, one study from Thailand reported ZEN concentrations of 0.96–55.6 µg/kg in cow feed, and low to middle concentrations of ISF (0.030–57.9 mg/kg) in cow feed [40]. Additionally, a study published in 2021 showed that ISF co-occurred with ZEN in pasture samples from Austria [170].

### 4.2. Spectrum 380^®^ Method

In most studies, occurrence data are limited to either myco- or phytoestrogens. In general, information on the co-occurrence of phytoestrogens is scare. BIOMIN has been conducting analyses to investigate the co-occurrence of feed contaminants including mycotoxins since 2004. Ten years later Spectrum 380^®^ was launched in cooperation with the Institute of Bioanalytics and Agro-Metabolomics of the University of Natural Resources and Life Sciences, Vienna (BOKU). Spectrum 380^®^ is a service to customers with the aim to monitor more than 700 mycotoxins and other secondary metabolites of bacteria and fungi, 300 pesticides and 150 veterinary drugs [192]. Since the inclusion of phytoestrogens in January 2019, 1694 feed samples were analyzed. These feed samples are either finished feed or feed components which will be mixed prior to use. Table 2, Table 3, Table 4 and Table 5 provide descriptive statistics for the occurrence of ISF, ZEN, and ZEN metabolites in feed designated for different animal species.

#### 4.2.1. Aquaculture Feed

Between January 2019 and April 2021, a total of 26 aquaculture feed samples from different countries were analyzed (Table 2) and 92% of those were classified as finished feed samples. ISF were detected in 96% of these samples. The individual ISF were present in 85%–96% of the feed samples (Table 2). Only GEN, DAI, and GLY and their glycosides were detected, with genistin and daidzin being the most prevalent (Table 2), suggesting that the major ISF source was soybean. Glucosides occurred in higher concentrations than their aglycons (Table 2). The mean and maximum concentrations were 7.91 and 37.6 mg/kg, respectively, for GEN, and 26.7 and 129 mg/kg, respectively, for its glucoside genistin. Slightly lower values were observed for DAI and its glucoside daidzin (Table 2).

In aqua feed, ZEN occurred in the medium to high µg/kg range with mean and maximum concentrations of 233 μg/kg and 1045 μg/kg, respectively. The prevalence of ZEN was high with detectable concentrations in 73% of the samples. The concentrations of phytoestrogens were at least one order of magnitude higher than those of ZEN (Table 2). For example, the ratio of medians was 0.04 for ZEN/DAI and 0.03 for ZEN/GEN. ISF and ZEN co-occurred in 58–69% of the samples.

#### 4.2.2. Cattle Feed

ISF were detected in 43% of cattle feed samples collected from January 2019 to April 2021 (*n* = 542). The prevalence of the individual ISF in cattle feed ranged from 2.6% to 31% (Table 3). In addition to the ISF that were detected in the aquaculture feed samples (Section 4.2.1; Table 2), biochanin A and formononetin were detected. Formononetin even reached higher mean and median concentrations than the other ISF (42.8 mg/kg and 13.9 mg/kg, respectively; Table 3). Feed for cattle often consist of different grasses, as well as clover species rich in biochanin A and formononetin, which could explain the high concentrations of these ISF in our samples.

Detectable levels of ZEN were present in 58% of cattle feed samples with mean, median, and maximum concentrations of 42.3 µg/kg, 11.1 µg/kg and 1305.0 µg/kg, respectively. ZEN metabolites (α-ZEL) and β-zearalenol (β-ZEL) were detected in 3% of the tested samples. Median concentrations of phytoestrogens were at least one order of magnitude higher than those of ZEN and its metabolites α-ZEL and β-ZEL (Table 3). The ratios of the median concentration of ZEN to the median concentration of each individual ISF were ≤0.02 (Table 3). ISF and ZEN co-occurred in 24% of cattle feed samples (percentage of samples co-contaminated with ZEN and individual ISF: 1.5–18.5%). Therefore, co-occurrence of ZEN and ISF was less frequently detected than in aquaculture (Table 2), pig (Section 4.2.3) and poultry (Section 4.2.4) feed samples.

#### 4.2.3. Pig Feed

More than 40% of the samples analyzed in this study were specified as pig feed (*n* = 862). ISF were detected in up to 45% of the pig feed samples, with daidzin and genistin as well as their aglycones being the most prevalent ones (Table 4). The detected mean, median, and maximum concentrations were 9.2, 4.7 and 140.0 mg/kg, respectively, for GEN and 63.3, 49.9 and 442.0 mg/kg, respectively, for its glucoside genistin; DAI and its glucoside daidzin were detected at similar but somewhat lower concentrations (Table 4). Biochanin A and formononetin were only present in 9% and 2% of samples, respectively, indicating that the major source of ISF in the pig feed samples analyzed in this study were soybeans.

ZEN occurred in the low to medium μg/kg range in most samples, with mean, median, and maximum concentrations of 115.0 μg/kg, 13.6 µg/kg and 9905.0 μg/kg, respectively (Table 4). The prevalence of ZEN was relatively high with detectable concentrations in 45% of the samples. ZEN metabolites α-ZEL and β-ZEL were detected in only 1.2% and 1.6% of the tested samples, respectively. ISF and ZEN co-occurred in 32% of the samples. Co-occurrence of ZEN with each individual ISF was detected in 1.5–30% of the samples (Table 4). Median ratios of ZEN/ISF did not exceed 0.07 (Table 4).

Pig feed was further divided into feed for ‘pig’ (*n* = 463), ‘pig-boar’ (*n* = 7), ‘pig-finisher’ (*n* = 10), ‘pig-grower’ (*n* = 33), ‘pig-piglet’ (*n* = 175) and ‘pig-sow’ (*n* = 172) (Tables S1–S6 are provided in the Supplementary Materials). The samples that were specified as ‘pig’ did not show a high incidence of either ISF (highest for daidzin 14%) or ZEN (16%) and its metabolites (Supplementary Table S1). However, the mean and median values for ISF were comparable with the values obtained for all pig feed samples (Table 4; Supplementary Tables S1–S6), whereas in the case of ZEN, the mean (318 µg/kg) exceeded the mean detected for all pig feed samples. The co-occurrence of ISF and ZEN for these samples was low, with the highest incidence of 6% for daidzin and ZEN. The situation was different for samples classified as ‘pig-piglet’, where ISF and ZEN were detected in up to 71% and 86% of samples, respectively. Mean, median and maximum concentrations were highest for genistin (64.6, 64.9 and 260 mg/kg), followed by daidzin and glycitin (Supplementary Table S5). The concentration of glycosides was 7–9 times higher compared to that of their aglycones, where the highest mean value was also observed for GEN, followed by DAI and GLY. The mean and maximum concentrations for ZEN were 43.1 µg/kg and 595 µg/kg. ZEN co-occurred with individual soy ISF in 60–62% of the samples (Supplementary Table S5). Soy ISF were even more prevalent in ‘pig-sow’ feed. Here, up to 80% of all samples contained soy ISF (Supplementary Table S1). Biochanin A und formononetin were present only in a small number of samples (Supplementary Table S6). Detectable levels of ZEN were present in 73% of the ‘pig-sow’ feed samples with mean, median and maximum concentrations of 100 µg/kg, 17.3 µg/kg and 3262 µg/kg, respectively.

The European Commission published guidance values for ZEN that should not be exceeded in certain feed commodities [36]. The mean values for the ‘pig-piglet’ and ‘pig-sow’ samples were below the guidance values for these two groups (100 and 250 µg/kg, respectively). Only the mean value of the feed classified as ‘pig’ exceeded the guidance values. Of all farm animals, young piglets are the most sensitive to ZEN regarding its effects on the reproduction [32]. As early as 1928, the occurrence of hyperestrogenism in swine, characterized by swelling and erythema of the vulva and uterine prolapse in gilts as well as atrophy of testicles and enlargement of nipples, could be associated with the consumption of ZEN contaminated cereals [193]. Although the feed samples declared as pig are not specified for any life stage, young piglets could be exposed to such high concentrations of ZEN, potentially adversely affecting their health.

#### 4.2.4. Poultry Feed

In total, 263 poultry feed samples were analyzed for the presence of ISF and ZEN and its metabolites (Table 5). Soy ISF were present in 88% of the feed samples (occurrence of individual ISF: 60%–81%; Table 5). Aglycones were again detected in lower concentrations by a factor of 7–10 compared to the respective glycosides (Table 5). The mean, median, and maximum concentrations were 10.4 mg/kg, 8.71 mg/kg and 102 mg/kg for GEN and 78.2 mg/kg, 60.4 mg/kg and 465 mg/kg for its glucoside genistin. The corresponding values for DAI and its glucoside daidzin were similar but somewhat lower compared to GEN and genistin (Table 5). Formononetin and biochanin A showed only low prevalence in poultry feed (0.8 and 5%, respectively). The prevalence of ZEN was high, with detectable concentrations in 67% of the samples and mean, median, and maximum concentrations of 53.1 µg/kg, 17.7 µg/kg and 873 µg/kg, respectively. Soy ISF and ZEN co-occurred in a high number of poultry samples (i.e., 63%; co-occurrence of individual ISF with ZEN: 49–60%). The ratio of median concentrations of ZEN/soy ISF were ≤0.007.

Similar to pig feed samples, poultry feed was further divided into subgroups: ‘poultry’ (*n* = 63), ‘poultry-breeder’ (*n* = 31), ‘poultry-broiler’ (*n* = 124) and ‘poultry-layer’ (*n* = 38) (the respective Tables S7–S11 are provided in the Supplementary Materials). For the different poultry feed subgroups, the distribution, mean and maximum values for both ISF and ZEN and its metabolite were similar when compared to all poultry feed samples (Supplementary Table S2).

## 5. Conclusions

ISF can cause both positive and negative effects in animals, depending on the species, the age and sex of the animals, and the dose and the frequency of exposure. Moderate concentrations of ISF, which occur naturally in feed, can have positive effects on growth performance, laying performance, and milk production. However, also negative effects on growth and laying performance have occasionally been observed. Furthermore, in ruminants, pigs, and fish, high concentrations of ISF were shown to negatively affect reproductive health.

Based on a literature review and analysis of 1694 feed samples with the Spectrum 380^®^ method, it is safe to say that both the prevalence and the concentrations of ISF are high in animal feed. The predominant ISF vary between feed destined for different animal species. Although formononetin is the dominant ISF in cattle, GEN and DAI are the most abundant ISF in feed destined for other species. ISF frequently co-occur with ZEN in animal feed. Same as ISF, ZEN can have a negative effect on reproductive health. It has already been reported that combinations of even small amounts of ISF and ZEN lead to an increased estrogenic effect when compared to the potency of the single substances in vitro [41]. Such an increase in estrogenic potency resulting from combinations of these two substance classes should be investigated further in the future. Clarification is needed whether co-occurrence of these estrogen-active compounds might result in a critical shift of the endocrine activity.

## Figures and Tables

**Table 1:** Occurrence data of ISF and ZEN and its metabolites in feed from literature of the last decade.

MYT/ISF	Food Commodity	Samples Tested	Positive Samples	Country	Detection Technique	Concentration Range (µg/kg)	Average (µg/kg)	LOD (µg/kg)	LOQ (µg/kg)	Mean Recovery	Reference
ZEN	Pig feed	20	6	China	UPLC-MS/MS	<LOQ–18.7	1.8	0.25 ng/mL	0.75 ng/mL	>90%	[171]
ZEN	Cattle feed	20	4	China	UPLC-MS/MS	<LOQ–14.4	1.4	0.25 ng/mL	0.75 ng/mL	>90%	[171]
ZEN	Chicken feed	20	5	China	UPLC-MS/MS	<LOQ–61.5	4.8	0.25 ng/mL	0.75 ng/mL	>90%	[171]
ZEN	Fish feed	11	11	Europe	HPLC	3–511	67.9	2.0	6.0	79%	[172]
ZEN	Pig feed	17	1	Korea	HPLC-MS/MS		124.8 (mg/kg)		3.1	106%	[173]
ZAL	Pig feed	17	2	Korea	HPLC-MS/MS	4.7–6.7 (mg/kg)		0.6	2	95%	[173]
β-ZAL	Pig feed	17	1	Korea	HPLC-MS/MS		3.1 (mg/kg)	0.3	1	104%	[173]
α-ZAL	Pig feed	17	2	Korea	HPLC-MS/MS	2.3–2.5 (mg/kg)		0.4	1.3	98%	[173]
α-ZAL	Chicken feed	13	2	Korea	HPLC-MS/MS	13.7–19.1 (mg/kg)		0.4	1.3	98%	[173]
β-ZAL	Cattle feed	14	1	Korea	HPLC-MS/MS		2.5	0.3	1	104%	[173]
ZEN	Poultry feed	9	1	Spane	UPLC-MS/MS			25	50	>100%	[174]
ZEN	Cattle feed	6	1	Spain	UPLC-MS/MS		52.2	25	50	>100%	[174]
ZEN	Sheep feed	17	3	Spain	UPLC-MS/MS	104.4–54.4		25	50	>100%	[174]
ZEN	Swine feed	20	2	Spain	UPLC-MS/MS			25	50	>100%	[174]
ZEN	Sow feed	15	15	Hungary	ELISA	18–35	21	17	51	>85%	[175]
ZEN	Boar feed	15	15	Hungary	ELISA	19–192	71	17	51	>85%	[175]
ZEN	Piglet feed	15	15	Hungary	ELISA	18–40	24.4	17	51	>85%	[175]
ZEN	Broiler feed	100	63	Thailand	LC-MS/MS	2.2–263.5	84.3	0.78	2	>93%	[176]
ZEN	Feed	466	386	Poland	HPLC-MS/MS	0.07–1113	18.6	0.07	0.2		[177]
ZEN	Pig feed (powder)	25	24	China	HPLC	10–835.4	272.1	1.5	10		[178]
ZEN	Pig feed (pellet)	90	73	China	HPLC	10–3346	634	1.5	10		[178]
ZEN	Duck feed	6	6	China	HPLC	10–2613.7	1718.3	1.5	10		[178]
ZEN	Maize	30	13	Poland	HPLC	n.d.–59.9	18.4	1			[179]
ZEN	Pig feed (pellet)	132	132	China	HPLC	10–4279.3	973.6	10	24		[180]
ZEN	Pig feed (powder)	427	425	China	HPLC	10–10,437.6	947.2	10	24		[180]
ZEN	Soybean meal	31	29	China	HPLC	10–6.9	4.2	10	24		[180]
ZEN	Corn bran	8	8	China	HPLC	10–13.5	7.3	10	24		[180]
ZEN	Lactation sow feed	13	13	China	HPLC	7.4–231	76	1.5	4	85%	[181]
ZEN	Gestating sow feed	10	10	China	HPLC	9.2–149	63	1.5	4	85%	[181]
ZEN	Grower feed	18	18	China	HPLC	7.1–150	59	1.5	4	85%	[181]
ZEN	Soybean meal	11	6	China	HPLC	LOQ–35.4	9	1.5	4	85%	[181]
ZEN	Feed, maize	1113	884	World	LC-MS/MS	>1–11,192		>1			[182]
ZEN	Poultry feed	20	17	Korea	HPLC	5.2–147.5	35	1.3	8	>75%	[183]
ZEN	Dairy cattle feed	40	24	South Africa	LC-QTOF-MS7MS	LOQ–28	2.8	0.04	0.12	>150%	[184]
α-ZEL	Dairy cattle feed	40	40	South Africa	LC-QTOF-MS7MS	1–13.2	4.8	0.19	0.63	>99%	[184]
β-ZEL	Dairy cattle feed	40	40	South Africa	LC-QTOF-MS7MS	0.7–4.7	2.4	0.19	0.64	>99%	[184]
ZEN	Complete pig feed	30	30	Norway	HPLC	<3–217	37				[185]
α-ZEL	Animal feed	77	5	Egypt	LC-MS/MS	LOQ–8		1.3	4.5	84%	[186]
β-ZEL	Animal feed	77	28	Egypt	LC-MS/MS	LOQ–60		1.2	3.5	87%	[186]
ZEN	Animal feed	77	71	Egypt	LC-MS/MS	LOQ–791		0.6	2.1	86%	[186]
ZEN	Finished feed	146	94	Africa	LC-MS/MS	LOQ–518					[187]
ZEN	Finished feed	301	173	South Africa	LC-MS/MS	LOQ–386					[187]
ZEN	Soy	30	24	Brazil	HPLC	LOQ–104	16.7	2	6	>99%	[188]
ZEN	Soybean meal	14	10	Pakistan	HPLC	0.15–120.9	18.9	0.05	0.15	>85%	[189]
ZEN	Poultry feed	11	9	Pakistan	HPLC	0.15–125.2	15.8	0.05	0.15	>85%	[189]
ZEN	Poultry feed	13	10	Pakistan	HPLC	0.15–118.4	19.6	0.05	0.15	>85%	[189]
ZEN	Cattle feed	174		Korea	HPLC-MS/MS		134.2	0.1–3	0.3–8	>96%	[190]
ZEN	Swine feed	160		Korea	HPLC-MS/MS		31.7	0.1–3	0.3–8	>96%	[190]
ZEN	Poultry feed	160		Korea	HPLC-MS/MS		37.9	0.1–3	0.3–8	>96%	[190]
ZEN	Cow mixed feed	34	28	Thailand	ESI-MS/MS	0.96–12.4	5.2	0.19		60%	[40]
ZEN	Cow concentrate	33	33	Thailand	ESI-MS/MS	2.5–55.6	24.3	0.19		60%	[40]
ZEN	Feed	61,413	27,559	Global		LOQ–105,000	55 *				[167]
ZEN	Finished feed	19,171	10,676	Global		LOQ–9432	41 *				[167]
ZEN	Maize	15,860	7992	Global		LOQ–16,495	77 *				[167]
ZEN	Maize silage	3735	1508	Global		LOQ–6239	84 *				[167]
ZEN	Soybean grains	1024	364	Global		LOQ–4336	43 *				[167]
ZEN	Soybean meal	1767	1072	Global		LOQ–3720	47 *				[167]
ZEN	Wheat	4925	1624	Global		LOQ–23,278	34 *				[167]
ZEN	Barley	3129	637	Global		LOQ–8952	25 *				[167]
ZEN	Rice	220	74	Global		LOQ–1530	60 *				[167]
DAI	Cow mixed feed	34	17	Thailand	ESI-MS/MS	1013–3759	2024	0.5		70%	[40]
Daidzin	Cow mixed feed	34	16	Thailand	ESI-MS/MS	30–15,030	5025	0.8		47%	[40]
GEN	Cow mixed feed	34	18	Thailand	ESI-MS/MS	790–4255	2447	0.8		85%	[40]
Genistin	Cow mixed feed	34	16	Thailand	ESI-MS/MS	104–20,106	6824	0.8		50%	[40]
GLY	Cow mixed feed	34	18	Thailand	ESI-MS/MS	129–1474	652	0.8		61%	[40]
Glycitin	Cow mixed feed	34	15	Thailand	ESI-MS/MS	190–3113	1406	0.8		56%	[40]
DAI	Cow concentrate	33	21	Thailand	ESI-MS/MS	1344–4720	2678	0.5		74%	[40]
Daidzin	Cow concentrate	33	21	Thailand	ESI-MS/MS	16,520–42,736	24,157	0.8		100%	[40]
GEN	Cow concentrate	33	21	Thailand	ESI-MS/MS	1319–4927	3056	0.8		107%	[40]
Genistin	Cow concentrate	33	21	Thailand	ESI-MS/MS	21,000–57,912	33,637	0.8		100%	[40]
GLY	Cow concentrate	33	21	Thailand	ESI-MS/MS	495–1686	1033	0.8		64%	[40]
Glycitin	Cow concentrate	33	21	Thailand	ESI-MS/MS	4539–13,648	8489	0.8		100%	[40]
Formononetin	Silage (clover)	3	3	Belgium	LC-MS/MS	3.1–11.3		0.16 ng/mL	0.53 ng/mL	>79%	[191]
Biochanin A	Silage (clover)	3	3	Belgium	LC-MS/MS	19–24.7		0.15 ng/mL	0.50 ng/mL	>47%	[191]
GEN	Silage (clover)	3	2	Belgium	LC-MS/MS	3.9–6.2		1.3 ng/mL	4.4 ng/mL	>92%	[191]
DAI	Silage (clover)	3	2	Belgium	LC-MS/MS	4.3–8.1		0.15 ng/mL	0.5 ng/mL	>80%	[191]
Formononetin	Silage (grass)	3	3	Belgium	LC-MS/MS	14.3–49.1		0.16 ng/mL	0.53 ng/mL	>79%	[191]
Biochanin A	Silage (grass)	3	2	Belgium	LC-MS/MS	6.8–24.1		0.15 ng/mL	0.50 ng/mL	>47%	[191]
GEN	Silage (grass)	3	3	Belgium	LC-MS/MS	3.1–5.7		1.3 ng/mL	4.4 ng/mL	>92%	[191]
DAI	Silage (grass)	3	2	Belgium	LC-MS/MS	2.8–10.8		0.15 ng/mL	0.5 ng/mL	>80%	[191]
Formononetin	Silage (cocksfoot)	8	8	Belgium	LC-MS/MS	444.8–687.6		0.16 ng/mL	0.53 ng/mL	>79%	[191]
Biochanin A	Silage (cocksfoot)	8	8	Belgium	LC-MS/MS	436.5–548.8		0.15 ng/mL	0.50 ng/mL	>47%	[191]
GEN	Silage (cocksfoot)	8	8	Belgium	LC-MS/MS	105.8–256.6		1.3 ng/mL	4.4 ng/mL	>92%	[191]
DAI	Silage (cocksfoot)	8	8	Belgium	LC-MS/MS	175.7–397.4		0.15 ng/mL	0.5 ng/mL	>80%	[191]
Daidzin	Soybean meal	6		Argentina	HPLC	494–785	596				[72]
DAI	Soybean meal	6		Argentina	HPLC	146–203	172				[72]
Genistin	Soybean meal	6		Argentina	HPLC	930–1274	1066				[72]
GEN	Soybean meal	6		Argentina	HPLC	69–110	82				[72]
Glycitin	Soybean meal	6		Argentina	HPLC	168–208	181				[72]
GLY	Soybean meal	6		Argentina	HPLC	152–279	190				[72]
Daidzin	Soybean meal	6		Brazil	HPLC	248–403	298				[72]
DAI	Soybean meal	6		Brazil	HPLC	60–143	122				[72]
Genistin	Soybean meal	6		Brazil	HPLC	551–704	607				[72]
GEN	Soybean meal	6		Brazil	HPLC	26–100	81				[72]
Glycitin	Soybean meal	6		Brazil	HPLC	116–168	142				[72]
GLY	Soybean meal	6		Brazil	HPLC	53–93	67				[72]
Daidzin	Soybean meal	6		USA	HPLC	234–257	326				[72]
DAI	Soybean meal	6		USA	HPLC	25–76	53				[72]
Genistin	Soybean meal	6		USA	HPLC	410–688	535				[72]
GEN	Soybean meal	6		USA	HPLC	7–41	24				[72]
Glycitin	Soybean meal	6		USA	HPLC	73–137	113				[72]
GLY	Soybean meal	6		USA	HPLC	71–137	96				[72]
ZEN	Pastures	18	9	Austria	LC-MS/MS	2.62–138	29.6				[170]
Biochanin A	Pastures	18	16	Austria	LC-MS/MS	62.1–20,650	7060				[170]
DAI	Pastures	18	15	Austria	LC-MS/MS	5.16–6110	926				[170]
Daidzin	Pastures	18	6	Austria	LC-MS/MS	15.8–543	167				[170]
GEN	Pastures	18	15	Austria	LC-MS/MS	28.4–17,550	2760				[170]
Genistin	Pastures	18	9	Austria	LC-MS/MS	14.6–1630	311				[170]
GLY	Pastures	18	15	Austria	LC-MS/MS	313–35,850	7470				[170]

MYT = mycotoxin; ISF = isoflavone; LOD = limit of detection; LOQ = limit of quantification; ZEN = Zearalenone; α-ZAL = α-Zearalanol; β-ZAL = β-Zearalanol; α-ZEL = α-Zearalenol; β-ZEL = β-Zearalenol; GEN = Genistein; DAI = Daidzein; GLY = Glycitein; values marked with * are reported as median values of positive samples.

**Table 2:** Occurrence of ISF and ZEN in aquaculture feed (Spectrum 380^®^). Please note that ZEN and ZEN-metabolite concentrations are provided in µg/kg whereas the ISF as feed constituents occur in higher concentrations and are reported in mg/kg (factor 1000 higher).

	Aqua Feed (*n* = 26)						
	DAI	Daidzin	GEN	Genistin	GLY	Glycitin	ZEN *
Positive samples (*n*)	22	25	22	25	23	24	19
Positive samples (%)	85%	96%	85%	96%	88%	92%	73%
Mean (mg/kg)	5.90	17.2	7.91	26.7	1.66	9.02	233
Maximum (mg/kg)	25.3	79.1	37.6	129	9.22	94.7	1045
Third quartile (mg/kg)	6.14	32.7	15.2	47.4	2.93	12.4	334
Median (mg/kg)	0.765	2.25	1.35	3.87	0.202	0.806	12.5
First quartile (mg/kg)	0.195	0.545	0.343	0.731	0.0298	0.335	3.82
Concentration ratio ZEN/ISF	0.04	0.01	0.03	0.01	0.14	0.03	
Co-occurrence of ISF and ZEN (*n*)	15	18	15	18	16	17	
Co-occurrence of ISF and ZEN (% of samples)	58	69	58	69	62	65	

Mycotoxins marked with * are reported in µg/kg; *n* = sample number; LOQ = limit of quantification; ISF = isoflavones; ZEN = Zearalenone; DAI = Daidzein; GEN = Genistein; GLY = Glycitein.

**Table 3:** Occurrence of ISF and ZEN and its metabolites in cattle feed (Spectrum 380^®^). Please note that ZEN and ZEN-metabolite concentrations are provided in µg/kg whereas the ISF as feed constituents occur in higher concentrations and are reported in mg/kg (factor 1000 higher).

	Cattle Feed (*n* = 542)										
	Biochanin A	DAI	Daidzin	Formononetin	GEN	Genistin	GLY	Glycitin	α-ZEL *	β-ZEL *	ZEN *
Positive samples (*n*)	118	144	146	14	170	140	93	88	16	16	317
Positive samples (%)	22%	27%	27%	3%	31%	26%	17%	16%	3%	3%	58%
Mean (mg/kg)	2.53	2.75	4.29	42.8	3.62	5.91	2.96	2.48	15.8	14.1	42.3
Maximum (mg/kg)	29.8	49.8	175	183	64.2	225	35.8	57.7	119	35.7	1305
Third quartile (mg/kg)	1.67	1.97	1.39	36.6	2.66	1.49	3.22	1.47	8.88	20.5	29.9
Median (mg/kg)	0.223	0.354	0.108	13.9	0.321	0.255	0.681	0.292	4.50	9.06	11.1
First quartile (mg/kg)	0.028	0.052	0.028	5.04	0.036	0.044	0.123	0.055	2.78	6.52	4.09
Concentration ratio ZEN/ISF	0.02	0.02	0.01	0.001	0.01	0.01	0.01	0.02			
Co-occurrence of ISF and ZEN (*n*)	60	85	86	8	100	78	57	58			
Co-occurrence of ISF and ZEN (% of samples)	11	16	16	1	18	14	11	11			

Mycotoxins marked with * are reported in µg/kg; LOQ = limit of quantification; Q1 = first quartile; Q3 = third quartile; ISF = isoflavone; ZEN = Zearalenone; α-ZEL = α-Zearalenol; β-ZEL = β-Zearalenol; DAI = Daidzein; GEN = Genistein; GLY = Glycitein.

**Table 4:** Occurrence of ISF and ZEN and its metabolites in pig feed (Spectrum 380^®^). Please note that ZEN and ZEN-metabolite concentrations are provided in µg/kg whereas the ISF as feed constituents occur in higher concentrations and are reported in mg/kg (factor 1000 higher).

	Pig Feed (*n* = 862)										
	Biochanin A	DAI	Daidzin	Formononetin	GEN	Genistin	GLY	Glycitin	α-ZEL *	β-ZEL *	ZEN *
Positive samples (*n*)	78	334	362	16	345	351	303	316	11	14	390
Positive samples (%)	9%	39%	42%	2%	40%	41%	35%	37%	1%	2%	45%
Mean (mg/kg)	4.18	5.98	40.3	1.66	9.15	63.3	2.89	18.5	25.0	10.7	115
Maximum (mg/kg)	316	61.9	358	15.7	140	442	163	118	105	36.0	9905
Third quartile (mg/kg)	0.018	8.01	60.0	0.109	11.4	86.9	2.34	21.6	34.8	13.8	40.0
Median (mg/kg)	0.008	3.17	34.0	0.056	4.69	49.9	0.795	12.7	7.84	6.99	13.6
First quartile (mg/kg)	0.003	1.05	2.36	0.031	1.23	6.91	0.369	4.10	4.87	5.52	4.96
Concentration ratio ZEN/ISF	0.027	0.019	0.003	0.069	0.013	0.002	0.040	0.006			
Co-occurrence of ISF and ZEN (*n*)	56	252	263	13	254	257	236	246			
Co-occurrence of ISF and ZEN (% of samples)	6	29	31	2	29	30	27	29			

Mycotoxins marked with * are reported in µg/kg; LOQ = limit of quantification; Q1 = first quartile; Q3 = third quartile; ISF = isoflavone; ZEN = Zearalenone; α-ZEL = α-Zearalenol; β-ZEL = β-Zearalenol; DAI = Daidzein; GEN = Genistein; GLY = Glycitein.

**Table 5:** Occurrence of ISF and ZEN and its metabolites in poultry feed (Spectrum 380^®^). Please note that ZEN and ZEN-metabolite concentrations are provided in µg/kg whereas the ISF as feed constituents occur in higher concentrations and are reported in mg/kg (factor 1000 higher).

	Poultry Feed (*n* = 263)										
	Biochanin A	DAI	Daidzin	Formononetin	GEN	Genistin	GLY	Glycitin	α-ZEL *	β-ZEL *	ZEN *
Positive samples (*n*)	13	176	205	2	191	212	159	168	10	18	177
Positive samples (%)	5%	67%	78%	1%	73%	81%	60%	64%	4%	7%	67%
Mean (mg/kg)	0.016	7.57	56.9	0.237	10.4	78.2	2.93	26.8	5.33	9.80	53.1
Maximum (mg/kg)	0.124	53.7	342	0.306	102	465	37.2	127	12.6	58.7	873
Third quartile (mg/kg)	0.011	10.2	86.4	0.271	14.6	131	3.32	37.9	7.45	6.13	50.4
Median (mg/kg)	0.007	6.46	39.3	0.237	8.71	60.4	1.86	19.4	4.24	2.74	17.7
First quartile (mg/kg)	0.004	1.98	0.790	0.202	1.12	0.453	0.67	6.49	1.75	1.66	6.95
Concentration ratio ZEN/ISF	3.2	0.007	0.001	0.2	0.005	0.001	0.018	0.002			
Co-occurrence of ISF and ZEN (*n*)	11	142	155	1	149	158	129	136			
Co-occurrence of ISF and ZEN (% of samples)	4	54	59	0	57	60	49	52			

Mycotoxins marked with * are reported in µg/kg; LOQ = limit of quantification; Q1 = first quartile; Q3 = third quartile; ISF = isoflavone; ZEN = Zearalenone; α-ZEL = α-Zearalenol; β-ZEL = β-Zearalenol; DAI = Daidzein; GEN = Genistein; GLY = Glycitein.

## Data Availability

Not applicable.

## Supplementary Materials

The following are available online at https://www.mdpi.com/article/10.3390/toxins13120836/s1, Table S1: Occurrence of ISF and ZEN in pig feed (Spectrum 380^®^), Table S2: Occurrence of ISF and ZEN in pig-boar feed (Spectrum 380^®^), Table S3: Occurrence of ISF and ZEN in pig-finisher feed (Spectrum 380^®^), Table S4: Occurrence of ISF and ZEN in pig-grower feed (Spectrum 380^®^), Table S5: Occurrence of ISF and ZEN in pig-piglet feed (Spectrum 380^®^), Table S6: Occurrence of ISF and ZEN in pig-sow feed (Spectrum 380^®^), Table S7: Occurrence of ISF and ZEN in poultry feed (Spectrum 380^®^), Table S8: Occurrence of ISF and ZEN in poultry-breeder feed (Spectrum 380^®^), Table S9: Occurrence of ISF and ZEN in poultry-broiler feed (Spectrum 380^®^), Table S10: Occurrence of ISF and ZEN in poultry-layer feed (Spectrum 380^®^), Table S11: Occurrence of ISF and ZEN in poultry-turkey feed (Spectrum 380^®^).
