# Supplementary material for: Isoflavones in Animals: Metabolism and Effects in Livestock and Occurrence in Feed
Source: Toxins (Basel). 2021 Nov 24;13(12):836. doi: 10.3390/toxins13120836 (PMC8705642; doi:10.3390/toxins13120836)
Supplement: Supplementary file 1 [file toxins-13-00836-s001.zip › toxins-1447110-supplementary.pdf]

Supplementary Materials: Isoflavones in Animals: Metabolism and Effects in Livestock and Occurrence in Feed

Dino Grgic, Elisabeth Varga, Barbara Novak, Anneliese Müller and Doris Marko

Content:

Table S1: Occurrence of ISF and ZEN in pig feed (Spectrum 380®). Page 2
Table S2: Occurrence of ISF and ZEN in pig - boar feed (Spectrum 380®). Page 3
Table S3: Occurrence of ISF and ZEN in pig - finisher feed (Spectrum 380®). Page 4
Table S4: Occurrence of ISF and ZEN in pig - grower feed (Spectrum 380®). Page 5
Table S5: Occurrence of ISF and ZEN in pig - piglet feed (Spectrum 380®). Page 6
Table S6: Occurrence of ISF and ZEN in pig - sow feed (Spectrum 380®). Page 7
Table S7: Occurrence of ISF and ZEN in poultry feed (Spectrum 380®). Page 8
Table S8: Occurrence of ISF and ZEN in poultry - breeder feed (Spectrum 380®). Page 9
Table S9: Occurrence of ISF and ZEN in poultry – broiler feed (Spectrum 380®). Page 10
Table S10: Occurrence of ISF and ZEN in poultry – layer feed (Spectrum 380®). Page 11
Table S11: Occurrence of ISF and ZEN in poultry - turkey feed (Spectrum 380®). Page 12

Toxins **2021**, *13*, 836. https://doi.org/10.3390/toxins13120836 www.mdpi.com/journal/toxins

Table S12: Occurrence of ISF and ZEN in pig feed (Spectrum 380®). Please note that ZEN and ZEN-metabolite concentrations are provided in µg/kg whereas the ISF as feed constituents occur in higher concentrations and are reported in mg/kg (factor 1000 higher).

	Pig (n=463)									
	Biochanin A	DAI	Daidzin	GEN	Genistin	GLY	Glycitin	α-ZEL*	β-ZEL*	ZEN*
Positive samples (n)	14	46	66	52	53	32	37	5	4	78
Positive samples (%)	3%	10%	14%	11%	11%	7%	8%	1%	1%	17%
Mean (mg/kg)	22.8	8.79	34.6	13.4	60.9	9.64	35.2	48.7	13.8	318
Maximum (mg/kg)	316	47.4	358	140	436	163	118	105	36.0	9905
Third quartile (mg/kg)	0.032	17.5	77.0	18.1	126	9.00	75.4	65.1	14.3	50.1
Median (mg/kg)	0.016	1.50	0.218	0.920	1.58	2.58	16.6	55.8	6.94	13.1
First quartile (mg/kg)	0.011	0.059	0.038	0.031	0.092	0.320	0.171	10.0	6.44	4.38
Concentration ratio ZEN/ISF	0.01	0.04	0.009	0.02	0.005	0.03	0.009			
Co-occurrence of ISF and ZEN (n)	4.0	16.0	26.0	16.0	20.0	11.0	16.0			
Co-occurrence of ISF and ZEN (% of samples)	0.9	3.5	5.6	3.5	4.3	2.4	3.5			

Mycotoxins marked with * are reported in µg/kg; LOQ = limit of quantification; Q1 = first quartile; Q3 = third quartile; ISF = isoflavone; ZEN = Zearalenone; α-ZEL = α-Zearalenol; β-ZEL = β-Zearalenol; DAI = Daidzein; GEN = Genistein; GLY = Glycitein.

Table S13: Occurrence of ISF and ZEN in pig - boar feed (Spectrum 380®). Please note that ZEN and ZEN-metabolite concentrations are provided in µg/kg whereas the ISF as feed constituents occur in higher concentrations and are reported in mg/kg (factor 1000 higher).

	Pig - Boar (n=7)							
	Biochanin A	DAI	Daidzin	GEN	Genistin	GLY	Glycitin	ZEN*
Positive samples (n)	2	7	7	7	7	6	7	5
Positive samples (%)	29%	100%	100%	100%	100%	86%	100%	71%
Mean (mg/kg)	0.013	9.31	78.4	17.7	101	4.59	40.9	4.09
Maximum (mg/kg)	0.015	32.3	208	58.9	247	15.3	83.6	12.1
Third quartile (mg/kg)	0.014	8.39	105	16.4	136	3.58	61.5	2.53
Median (mg/kg)	0.013	7.04	49.5	15.3	70.3	2.95	39.2	2.36
First quartile (mg/kg)	0.012	4.46	40.0	8.50	58.0	2.33	20.2	2.27
Concentration ratio ZEN/ISF	0.3	0.0004	0.0001	0.0002	0.00004	0.0009	0.0001	
Co-occurrence of ISF and ZEN (n)	0	5	5	5	5	4	5	
Co-occurrence of ISF and ZEN (% of samples)	0	71	71	71	71	57	71	

Mycotoxins marked with * are reported in µg/kg; LOQ = limit of quantification; Q1 = first quartile; Q3 = third quartile; ISF = isoflavone; ZEN = Zearalenone; DAI = Daidzein; GEN = Genistein; GLY = Glycitein.

Table S14: Occurrence of ISF and ZEN in pig - finisher feed (Spectrum 380®). Please note that ZEN and ZEN-metabolite concentrations are provided in µg/kg whereas the ISF as feed constituents occur in higher concentrations and are reported in mg/kg (factor 1000 higher).

	Pig - Finisher (n=10)							
	Biochanin A	DAI	Daidzin	GEN	Genistin	GLY	Glycitin	ZEN*
Positive samples (n)	1	8	8	10	10	8	8	9
Positive samples (%)	10%	80%	80%	100%	100%	80%	80%	90%
Mean (mg/kg)	0.022	12.4	24.8	13.4	26.7	2.21	4.71	18.5
Maximum (mg/kg)	0.022	21.1	92.1	28.9	120	4.47	20.2	62.2
Third quartile (mg/kg)	0.022	16.3	46.4	21.4	45.0	3.00	6.34	14.8
Median (mg/kg)	0.022	12.4	0.86	11.6	1.31	2.44	0.170	7.48
First quartile (mg/kg)	0.022	7.21	0.35	7.80	0.577	0.948	0.137	3.06
Concentration ratio ZEN/ISF	0.830	0.001	0.001	0.001	0.001	0.008	0.004	
Co-occurrence of ISF and ZEN (n)	7	9	9	9	9	7	7	
Co-occurrence of ISF and ZEN (% of samples)	70	90	90	90	90	70	70	

Mycotoxins marked with * are reported in µg/kg; LOQ = limit of quantification; Q1 = first quartile; Q3 = third quartile; ISF = isoflavone; ZEN = Zearalenone; DAI = Daidzein; GEN = Genistein; GLY = Glycitein.

Table S15: Occurrence of ISF and ZEN in pig - grower feed (Spectrum 380®). Please note that ZEN and ZEN-metabolite concentrations are provided in µg/kg whereas the ISF as feed constituents occur in higher concentrations and are reported in mg/kg (factor 1000 higher).

	Pig - Grower (n=33)								
	Biochanin A	DAI	Daidzin	Formononetin	GEN	Genistin	GLY	Glycitin	ZEN*
Positive samples (n)	3	22	20	1	21	20	17	18	22
Positive samples (%)	9%	67%	61%	3%	64%	61%	52%	55%	67%
Mean (mg/kg)	0.216	5.41	32.3	0.241	8.85	47.6	8.66	19.7	28.2
Maximum (mg/kg)	0.636	24.6	111	0.241	34.9	175	121	96.3	37.3
Third quartile (mg/kg)	0.324	8.23	44	0.241	15.7	63.4	3.33	18.1	163
Median (mg/kg)	0.011	3.81	30.0	0.241	4.86	43.1	1.59	12.8	38.3
First quartile (mg/kg)	0.006	0.206	8.37	0.241	0.914	11.1	0.385	8.29	17.4
Concentration ratio ZEN/ISF	0.13	0.005	0.001	0.12	0.003	0.001	0.003	0.001	
Co-occurrence of ISF and ZEN (n)	1	15	13	0	14	12	10	11	
Co-occurrence of ISF and ZEN (% of samples)	3	45	39	0	42	36	30	33	

Mycotoxins marked with * are reported in µg/kg; LOQ = limit of quantification; Q1 = first quartile; Q3 = third quartile; ISF = isoflavone; ZEN = Zearalenone; DAI = Daidzein; GEN = Genistein; GLY = Glycitein.

Table S16: Occurrence of ISF and ZEN in pig - piglet feed (Spectrum 380®). Please note that ZEN and ZEN-metabolite concentrations are provided in µg/kg whereas the ISF as feed constituents occur in higher concentrations and are reported in mg/kg (factor 1000 higher).

	Pig - Piglet (n=175)									
	Biochanin A	DAI	Daidzin	Formononetin	GEN	Genistin	GLY	Glycitin	β-ZEL*	ZEN*
Positive samples (n)	33	120	125	12	123	123	113	120	1	151
Positive samples (%)	19%	69%	71%	7%	70%	70%	65%	69%	1%	86%
Mean (mg/kg)	0.039	5.45	47.1	1.37	9.07	64.6	2.07	16.3	23.8	43.1
Maximum (mg/kg)	0.614	23.6	118	15.7	36.9	160	24.9	86.0	23.8	595
Third quartile (mg/kg)	0.011	8.42	72.9	0.062	12.4	94.2	2.16	21.6	23.8	35.2
Median (mg/kg)	0.004	3.90	41.4	0.052	6.71	64.9	0.986	14.9	23.8	12.6
First quartile (mg/kg)	0.003	2.29	28.2	0.031	3.48	40.7	0.594	8.56	23.8	6
Concentration ratio ZEN/ISF	1.1	0.008	0.001	0.03	0.005	0.001	0.02	0.003		
Co-occurrence of ISF and ZEN (n)	30	108	108	11	109	106	105	107		
Co-occurrence of ISF and ZEN (% of samples)	17	62	62	6	62	61	60	61		

Mycotoxins marked with * are reported in µg/kg; LOQ = limit of quantification; Q1 = first quartile; Q3 = third quartile; ISF = isoflavone; ZEN = Zearalenone; β-ZEL = β-Zearalenol; DAI = Daidzein; GEN = Genistein; GLY = Glycitein.

Table S17: Occurrence of ISF and ZEN in pig - sow feed (Spectrum 380®). Please note that ZEN and ZEN-metabolite concentrations are provided in µg/kg whereas the ISF as feed constituents occur in higher concentrations and are reported in mg/kg (factor 1000 higher).

	Pig - Sow (n=172)										
	Biochanin A	DAI	Daidzin	Formononetin	GEN	Genistin	GLY	Glycitin	α-ZEL*	β-ZEL*	ZEN*
Positive samples (n)	25	131	136	3	132	138	127	126	6	9	125
Positive samples (%)	15%	76%	79%	2%	77%	80%	74%	73%	3%	5%	73%
Mean (mg/kg)	0.181	5.00	36.9	3.30	6.82	66.0	1.12	15.2	5.31	7.95	100
Maximum (mg/kg)	2.89	61.9	354	9.83	91.2	442	10.2	101	13.8	15.0	3262
Third quartile (mg/kg)	0.019	4.44	46.9	4.95	6.20	70.3	1.13	17.7	4.92	13.3	42.3
Median (mg/kg)	0.008	2.41	29.6	0.061	2.92	40.5	0.499	9.85	4.87	6.96	17.3
First quartile (mg/kg)	0.002	0.828	8.55	0.040	1.31	11.1	0.194	3.61	2.59	3.46	6.20
Concentration ratio ZEN/ISF	0.6	0.02	0.003	0.03	0.01	0.002	0.09	0.007			
Co-occurrence of ISF and ZEN (n)	20	6	0	0	19	6	0	0			
Co-occurrence of ISF and ZEN (% of samples)	12	3	0	0	11	3	0	0			

Mycotoxins marked with * are reported in µg/kg; LOQ = limit of quantification; Q1 = first quartile; Q3 = third quartile; ISF = isoflavone; ZEN = Zearalenone; α-ZEL = α-Zearalenol; β-ZEL = β-Zearalenol; DAI = Daidzein; GEN = Genistein; GLY = Glycitein.

Table S18: Occurrence of ISF and ZEN in poultry feed (Spectrum 380®). Please note that ZEN and ZEN-metabolite concentrations are provided in µg/kg whereas the ISF as feed constituents occur in higher concentrations and are reported in mg/kg (factor 1000 higher).

	Poultry (n=63)									
	Biochanin A	DAI	Daidzin	GEN	Genistin	GLY	Glycitin	α-ZEL*	β-ZEL*	ZEN*
Positive samples (n)	2	42	50	45	49	37	38	2	1	37
Positive samples (%)	3%	67%	79%	71%	78%	59%	60%	3%	2%	59%
Mean (mg/kg)	0.003	9.62	62.8	12.4	95.4	3.24	45.7	9.23	55.8	42.8
Maximum (mg/kg)	0.004	29.4	315	38.7	449	9.32	127	11.5	55.8	486
Third quartile (mg/kg)	0.003	15.4	93.9	21.0	143	4.97	79.9	10.4	55.8	46.9
Median (mg/kg)	0.003	10.9	65.6	12.3	95.4	2.79	41.8	9.23	55.8	25.9
First quartile (mg/kg)	0.002	0.174	0.252	0.115	0.281	0.911	3.05	8.09	55.8	7.02
Concentration ratio ZEN/ISF	16	0.004	0.001	0.003	0.0004	0.013	0.001			
Co-occurrence of ISF and ZEN (n)	1.0	30.0	33.0	31.0	33.0	28.0	29.0			
Co-occurrence of ISF and ZEN (% of samples)	1.6	47.6	52.4	49.2	52.4	44.4	46.0			

Mycotoxins marked with * are reported in µg/kg; LOQ = limit of quantification; Q1 = first quartile; Q3 = third quartile; ISF = isoflavone; ZEN = Zearalenone; α-ZEL = α-Zearalenol; β-ZEL = β-Zearalenol; DAI = Daidzein; GEN = Genistein; GLY = Glycitein.

Table S19: Occurrence of ISF and ZEN in poultry - breeder feed (Spectrum 380®). Please note that ZEN and ZEN-metabolite concentrations are provided in µg/kg whereas the ISF as feed constituents occur in higher concentrations and are reported in mg/kg (factor 1000 higher).

	Poultry -Breeder (n=31)								
	DAI	Daidzin	GEN	Genistin	GLY	Glycitin	α-ZEL*	β-ZEL*	ZEN*
Positive samples (n)	18	21	20	25	15	16	3	4	17
Positive samples (%)	58%	68%	65%	81%	48%	52%	10%	13%	55%
Mean (mg/kg)	9.4	61.2	15.8	76.0	5.91	18.4	3.22	6.11	105
Maximum (mg/kg)	53.7	342	102	465	37.2	93.3	4.97	14.5	724
Third quartile (mg/kg)	7.19	46.2	12.5	76.3	2.28	25.5	4.24	8.58	120
Median (mg/kg)	4.23	29.3	9.62	18.4	1.76	7.23	3.50	4.41	38.0
First quartile (mg/kg)	1.88	0.803	1.43	0.206	0.391	1.51	2.35	1.94	10.1
Concentration ratio ZEN/ISF	0.01	0.002	0.007	0.001	0.02	0.006			
Co-occurrence of ISF and ZEN (n)	12	14	13	14	9	10			
Co-occurrence of ISF and ZEN (% of samples)	39	45	42	45	29	32			

Mycotoxins marked with * are reported in µg/kg; LOQ = limit of quantification; Q1 = first quartile; Q3 = third quartile; ISF = isoflavone; ZEN = Zearalenone; α-ZEL = α-Zearalenol; β-ZEL = β-Zearalenol; DAI = Daidzein; GEN = Genistein; GLY = Glycitein.

Table S20: Occurrence of ISF and ZEN in poultry – broiler feed (Spectrum 380®). Please note that ZEN and ZEN-metabolite concentrations are provided in µg/kg whereas the ISF as feed constituents occur in higher concentrations and are reported in mg/kg (factor 1000 higher).

	Poultry - Broiler (n=124)										
	Biochanin A	DAI	Daidzin	Formononetin	GEN	Genistin	GLY	Glycitin	α-ZEL*	β-ZEL*	ZEN*
Positive samples (n)	11	89	100	2	96	101	84	87	3	8	91
Positive samples (%)	9%	72%	81%	2%	77%	81%	68%	70%	2%	6%	73%
Mean (mg/kg)	0.019	7.45	61.8	0.237	10.2	83.6	2.78	24.0	1.63	2.66	44.8
Maximum (mg/kg)	0.124	34.2	216	0.306	38.5	292	18.9	100	2.27	5.83	873
Third quartile (mg/kg)	0.012	9.86	90.4	0.271	14.7	119	3.02	34.7	1.92	3.60	39.7
Median (mg/kg)	0.009	7.15	43.0	0.237	9.39	66.0	1.94	23.6	1.57	1.73	14.0
First quartile (mg/kg)	0.006	3.55	29.0	0.202	4.53	40.9	1.24	7.68	1.32	1.35	7.46
Concentration ratio ZEN/ISF	2.4	0.006	0.001	0.2	0.004	0.001	0.02	0.002			
Co-occurrence of ISF and ZEN (n)	10	77	79	1	80	82	71	73			
Co-occurrence of ISF and ZEN (% of samples)	8	62	64	1	65	66	57	59			

Mycotoxins marked with * are reported in µg/kg; LOQ = limit of quantification; Q1 = first quartile; Q3 = third quartile; ISF = isoflavone; ZEN = Zearalenone; α-ZEL = α-Zearalenol; β-ZEL = β-Zearalenol; DAI = Daidzein; GEN = Genistein; GLY = Glycitein.

Table S21: Occurrence of ISF and ZEN in poultry – layer feed (Spectrum 380®). Please note that ZEN and ZEN-metabolite concentrations are provided in µg/kg whereas the ISF as feed constituents occur in higher concentrations and are reported in mg/kg (factor 1000 higher).

	Poultry - Layer (n=38)								
	DAI	Daidzin	GEN	Genistin	GLY	Glycitin	α-ZEL*	β-ZEL*	ZEN*
Positive samples (n)	23	29	26	32	19	23	2	5	27
Positive samples (%)	61%	76%	68%	84%	50%	61%	5%	13%	71%
Mean (mg/kg)	2.92	25.0	3.89	33.3	0.867	10.7	10.1	15.0	70.8
Maximum (mg/kg)	15.0	184	21.3	280	6.29	101	12.6	58.7	389
Third quartile (mg/kg)	3.14	31.2	4.99	44.0	0.667	9.36	11.4	6.23	106
Median (mg/kg)	2.15	13.7	2.83	12.6	0.463	4.41	10.1	5.67	27.8
First quartile (mg/kg)	0.587	0.387	0.152	0.370	0.155	0.534	8.86	2.48	5.37
Concentration ratio ZEN/ISF	0.02	0.003	0.02	0.002	0.08	0.007			
Co-occurrence of ISF and ZEN (n)	20	25	22	25	18	21			
Co-occurrence of ISF and ZEN (% of samples)	53	66	58	66	47	55			

Mycotoxins marked with * are reported in µg/kg; LOQ = limit of quantification; Q1 = first quartile; Q3 = third quartile; ISF = isoflavone; ZEN = Zearalenone; α-ZEL = α-Zearalenol; β-ZEL = β-Zearalenol; DAI = Daidzein; GEN = Genistein; GLY = Glycitein.

Table S22: Occurrence of ISF and ZEN in poultry - turkey feed (Spectrum 380®). Please note that ZEN and ZEN-metabolite concentrations are provided in µg/kg whereas the ISF as feed constituents occur in higher concentrations and are reported in mg/kg (factor 1000 higher).

	Poultry - Turkey (n=7)						
	DAI	Daidzin	GEN	Genistin	GLY	Glycitin	ZEN*
Positive samples (n)	4	5	4	5	4	4	4
Positive samples (%)	57%	71%	57%	71%	57%	57%	57%
Mean (mg/kg)	7.38	67.9	10.3	98.7	1.65	31.9	10.7
Maximum (mg/kg)	18.6	169	25.5	193	4.55	56.1	22.0
Third quartile (mg/kg)	8.99	96.3	11.5	191	1.79	47.5	17.6
Median (mg/kg)	4.89	42.7	6.75	70.6	0.867	30.4	9.80
First quartile (mg/kg)	3.29	32.1	5.58	38.7	0.723	14.7	2.88
Concentration ratio ZEN/ISF	0.001	0.0002	0.001	0.0001	0.006	0.0003	
Co-occurrence of ISF and ZEN (n)	3	3	3	3	3	3	
Co-occurrence of ISF and ZEN (% of samples)	50	50	50	50	50	50	

Mycotoxins marked with * are reported in µg/kg; LOQ = limit of quantification; Q1 = first quartile; Q3 = third quartile; ISF = isoflavone; ZEN = Zearalenone; α-ZEL = α-Zearalenol; β-ZEL = β-Zearalenol; DAI = Daidzein; GEN = Genistein; GLY = Glycitein.
